# Modeled Sources, Transport, and Accumulation of Dissolved Solids in Water Resources of the Southwestern United States^1^

**DOI:** 10.1111/j.1752-1688.2011.00579.x

**Published:** 2011-10

**Authors:** David W Anning

**Keywords:** dissolved solids, salinity, streams, basin-fill aquifers, transport, accumulation, SPARROW

## Abstract

**Abstract:**

Information on important source areas for dissolved solids in streams of the southwestern United States, the relative share of deliveries of dissolved solids to streams from natural and human sources, and the potential for salt accumulation in soil or groundwater was developed using a SPAtially Referenced Regressions On Watershed attributes model. Predicted area-normalized reach-catchment delivery rates of dissolved solids to streams ranged from <10 (kg/year)/km^2^ for catchments with little or no natural or human-related solute sources in them to 563,000 (kg/year)/km^2^ for catchments that were almost entirely cultivated land. For the region as a whole, geologic units contributed 44% of the dissolved-solids deliveries to streams and the remaining 56% of the deliveries came from the release of solutes through irrigation of cultivated and pasture lands, which comprise only 2.5% of the land area. Dissolved-solids accumulation is manifested as precipitated salts in the soil or underlying sediments, and (or) dissolved salts in soil-pore or sediment-pore water, or groundwater, and therefore represents a potential for aquifer contamination. Accumulation rates were <10,000 (kg/year)/km^2^ for many hydrologic accounting units (large river basins), but were more than 40,000 (kg/year)/km^2^ for the Middle Gila, Lower Gila-Agua Fria, Lower Gila, Lower Bear, Great Salt Lake accounting units, and 247,000 (kg/year)/km^2^ for the Salton Sea accounting unit.

## Introduction

**FIGURE 1:**
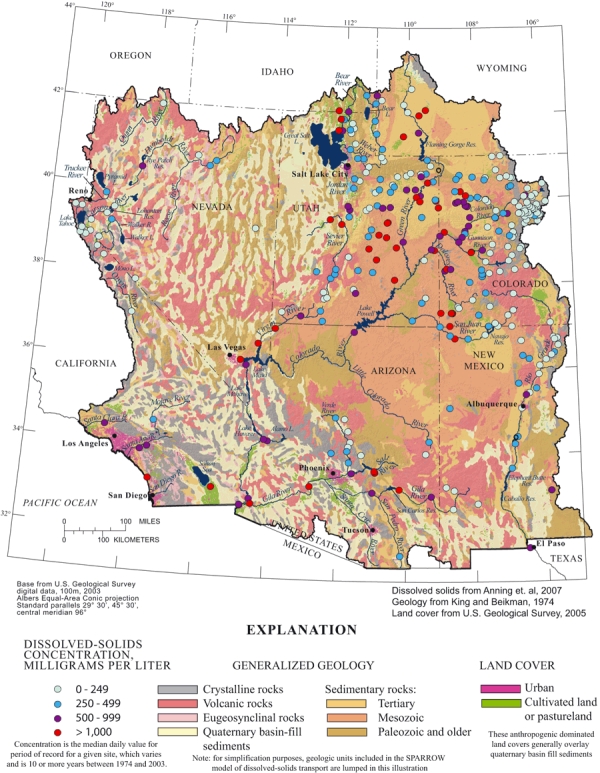
Median Daily Dissolved-Solids Concentrations at 315 Surface-Water Quality Monitoring Sites, Generalized Geology, and Land Cover in the Southwestern United States.

The location and extent of economic and cultural activities in the southwestern United States (U.S.) (the Southwest) are dependent in part on the availability and quality of water. Residents rely on diversions from the Colorado River, the Rio Grande, their tributaries, or the many smaller river systems that drain internally within the Great Basin (provinces shown in Figure S1), or drain to the Pacific Coast in southern California (Figure 1). Where and when surface water is not available, groundwater, generally from basin-fill aquifers, is used as a source of supply. In many areas of the Southwest, high concentrations of dissolved solids degrade a water supply's suitability for use. In response to this water-quality issue, the U.S. Geological Survey's (USGS) National Water-Quality Assessment (NAWQA) Program completed a regional study to characterize dissolved-solids conditions in the basin-fill aquifers and streams of the 1.3 million km^2^ Southwest region, and to understand how natural and human factors affect those conditions (Anning *et al.*, 2007). This article describes the application of a spatially referenced regression model completed as part of that study and improves the understanding of the sources and transport of dissolved solids in streams of the Southwest, and the potential for salt accumulation in basin-fill aquifers.

All surface water and groundwater naturally contain dissolved solids as a result of the weathering and dissolution of minerals in soils and geologic formations. Major ions, such as bicarbonate, calcium, chloride, magnesium, potassium, silica, sodium, and sulfate, constitute most of the dissolved solids in water and are collectively an indicator of salinity. Use of water typically increases its dissolved-solids concentrations. For example, detergents and softeners are commonly added to water during domestic use and may be returned to streams at wastewater-treatment plant outfalls. Irrigation accelerates the weathering process and release of native solutes from soils by applying more water to them than they naturally receive from precipitation under the arid to semiarid climate (Tanji, 1990). Excess irrigation water not evaporated or transpired can leach additional salts from soils as it drains back to the stream through surface or subsurface paths. In large basins with little natural recharge to flush the groundwater system, dissolved solids carried in the excess irrigation water may accumulate in the soil, and if infiltration is great enough, enter the groundwater and accumulate in the aquifer due to long residence times.

While some amount of constituents that comprise the dissolved solids is needed for plant and animal growth and for agricultural, domestic, municipal, and industrial purposes, excessively elevated concentrations affect aquatic ecosystems and water users through salt accumulation in soils, encrustment or corrosion of metallic surfaces, and altered osmotic conditions in living tissues (Ayers and Westcot, 1994; Chapman *et al.*, 2000; Scannell and Jacobs, 2001; Bureau of Reclamation, 2005). In the U.S. portion of the Colorado River Basin, the damage costs to agricultural, municipal, and industrial users of water high in dissolved-solids concentrations are estimated to range from US$500 million to US$750 million per year (Bureau of Reclamation, 2005). Several salinity-control projects were constructed in the Colorado River Basin to improve or prevent further degradation in the quality of Colorado River water for use by the U.S. and Mexico (Bureau of Reclamation, 2005). These salinity-control projects have included canal lining, lateral piping, on-farm irrigation control, irrigation drainage, pumping of groundwater, well plugging, vegetation management, and land retirement. As of 2004, it is estimated that the projects in operation reduced salt loading to the Colorado River by about 890,000 kg of dissolved solids per year (Colorado River Basin Salinity Control Forum, 2005), which is about 13% of the load in releases from Lake Mead. In municipal areas where salinity-control projects for source control are more difficult to achieve, concentrations of dissolved solids in brackish water supplies are reduced through costly water-treatment processes, such as reverse osmosis.

For the Southwest, a regional-scale understanding of dissolved-solids sources, transport, and accumulation is important because much of the water and dissolved solids originate from sources hundreds of kilometers from the place of water use. The primary objectives for this study were to (1) broaden the understanding of the geographic distribution of the sources, transport, and accumulation of dissolved solids for use by land and water managers to identify and develop salinity-control measures; and (2) increase the understanding of the relative importance of solute deliveries to streams from natural sources and those resulting from human activities, to provide land and water managers insight to the potential of how much loading can and cannot be controlled.

Frequently, the focus of regional-scale contaminant transport studies is to determine the sources and loadings of the contaminants to a single receiving surface-water body, such as nutrient loading to the Gulf of Mexico (Alexander *et al.*, 2008). For this study, however, several such receptors are dispersed across the Southwest, including many streams diverted for municipal or agricultural uses, and aquifers in which dissolved solids accumulate from irrigation seepage or from streamflow infiltration.

## Previous Investigations

Several studies have investigated dissolved-solids conditions within parts of the Southwest over the past century; however, most considered only selected small river basins or focused on temporal trends in concentrations. While no studies have investigated dissolved-solids sources, transport, or accumulation for the entire Southwest, a few have investigated these factors in selected areas.

Notable studies that investigated dissolved-solids sources for larger basins include the work of Mills (2003), Iorns *et al.* (1965), and Kenney *et al.* (2009). Mills (2003) investigated the causes of a 50-fold increase in dissolved solids concentrations in the Rio Grande as it flows from its headwaters to the U.S.-Mexico border. Mills (2003) found that the increase of chloride concentration in that reach resulted from inflow from natural tributaries (25% of the increase), seepage of deep-origin sedimentary-basin brines (37%), inflow of wastewater-treatment plant effluent (26%), and Elephant Butte Reservoir dynamic water-level fluctuations (9%).

Iorns *et al.* (1965) determined dissolved-solids concentrations and annual loads for several locations throughout the Upper Colorado River Basin, and used a nonstatistical approach to determine the portions of the dissolved-solids load resulting from geologic sources and irrigated lands. Iorns *et al.* (1965) determined yields of dissolved solids for 1957 from 21 areas that make up 41% of the irrigated lands in the Upper Colorado River Basin and found that those yields ranged from 22,000 to 1,240,000 (kg/year)/km^2^. Basin wide, Iorns *et al.* (1965) determined that the area-weighted average yield of dissolved solids for irrigated lands was 561,000 (kg/year)/km^2^.

Kenney *et al.* (2009) used the SPAtially Referenced Regressions On Watershed attributes (SPARROW) model described in this article and in Anning *et al.* (2007) as a framework from which to develop a finer-scale SPARROW model specific to the Upper Colorado River Basin. The Kenney *et al.*’s (2009) model used a finer river-reach network and finer spatial-scale data for geologic-source and irrigation-source variables than Anning *et al.* (2007), and distinguished dissolved-solids deliveries to streams from irrigated areas underlain by three different types of soils. Kenney *et al.* (2009) attributed 57% of the dissolved-solids load in the Colorado River at Lees Ferry, Arizona (about 25 km downstream from Lake Powel as shown in Figure 1) as coming from geologic sources, and 43% from irrigated lands, results that are comparable to estimates by Iorns *et al.* (1965) of 60% from geologic sources and 40% from irrigated lands.

Notable studies that focused on dissolved-solids accumulation for larger basins include the work of Anning (2003), Central Arizona Salinity Study (2003), and Metropolitan Water District of Southern California and U.S. Department of Interior, Bureau of Reclamation (1999). Anning (2003) and Central Arizona Salinity Study (2003) investigated salt accumulation in central Arizona using a mass-balance approach and monitoring data, and both studies found about 1 million tons of salt accumulated annually in that area. Metropolitan Water District of Southern California and U.S. Department of Interior, Bureau of Reclamation (1999) did a mass balance for the Southern California Coastal basins and found nearly 600,000 kg of dissolved solids accumulated annually in those basins. All three studies found that imported surface water was an important source of dissolved-solids loads transported into the area.

## Approach and Methods

Important source areas and accumulation areas for dissolved solids in water in the Southwest were evaluated through a mass balance of contributions and losses within river systems (streams, lakes, and reservoirs) of hydrologic accounting units. Hydrologic accounting units are river basins defined across the U.S. (Seaber *et al.*, 1987), and average about 41,000 km^2^ each in the Southwest. Contributions of dissolved solids to accounting unit river systems include: inflows, *L*_in_, the annual loads delivered to streams from upstream accounting unit streams; internal deliveries, *I*_del_, the annual loads delivered to accounting unit streams from internal sources of their watersheds; and imports, *T*_imp_, the annual loads conveyed into accounting unit streams or water-supply systems from transbasin imported water. Losses of dissolved solids from the accounting unit surface waters include: outflows, *L*_out_, the annual load that flows out of accounting unit streams to downstream accounting units; internal accumulation, *I*_acc_, the annual load removed from accounting unit streams that are retained and accumulate internally within accounting unit watersheds; and exports, *T*_exp_, the annual load conveyed out of accounting unit streams or water-supply systems to other areas through transbasin exported water. The following mass-balance equation for an accounting unit's river system shows the relation between contributions, losses, and change in river system storage, Δ*S*, of dissolved-solids mass (all terms have units of kg/year): 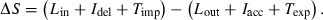
(1)

A subtlety to distinguish is that, because the control volume for Equation (1) is the accounting unit river system, Δ*S* is the storage within the streams and reservoirs of the river system, and *I*_acc_ results in storage of dissolved solids within the reach catchment but outside of the river system. Studies of mass transport often focus on stream yields, which are computed as outflow, *L*_out_, divided by the drainage area. By moving *L*_out_ to the left side of Equation (1) and assuming Δ*S* equals zero, it can be seen that, for an accounting unit, yield is a function of inflows, internal deliveries, imports, internal accumulation, and exports. To determine important source areas and accumulation areas of dissolved solids, however, the focus must not be on yields but rather on internal deliveries and internal accumulation. For comparison across accounting units, area-normalized values for *I*_del_ and *I*_acc_ were computed and referred to hereinafter as “delivery rates” and “accumulation rates,” respectively. While these rates represent an average value for the accounting unit as a whole, some parts will have higher rates and other parts will have lower rates.

Quantification of terms for the mass-balance equation was facilitated through a SPARROW model (Smith *et al.*, 1997; Schwarz *et al.*, 2006). Values for *L*_in_ and *L*_out_ were taken from stream-load predictions of dissolved solids from the SPARROW model at the inlet(s) and outlet(s) of each accounting unit. Loads for *T*_imp_ and *T*_exp_ were determined from reported annual diversions and from dissolved-solids concentrations of those waters, and then aggregated for each accounting unit. Further description and a summary of estimates for *T*_imp_ and *T*_exp_ are available in Anning *et al.* (2007). Values for *I*_del_ were determined as the sum of the predicted deliveries to streams from the SPARROW model results for all sources within all catchments that comprise the accounting unit. The SPARROW model has loss terms that reflect internal accumulation processes and exports; however at the time of study, output from the model did not allow for direct separation of *I*_acc_ and *T*_exp_. For this reason, *I*_acc_ for accounting units was determined as the residual of the sum of contributions of dissolved solids minus the outflow and exports of dissolved solids, and assuming that Δ*S* was zero: 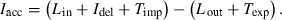
(2)

Use of this calculation of *I*_acc_ has the benefit of a zero-value residual for the mass balance. The errors from each term accumulate in the calculation, however, and increase the uncertainty for the internal loss estimate. If *T*_imp_ and *T*_exp_ are known, there are advantages to using the SPARROW model results to define *L*_in_, *L*_out_, *I*_del_, and *I*_acc_ in Equation (1), compared to using stream-load monitoring data alone. One advantage is that estimates for *L*_in_ and *L*_out_ can be obtained for cases in which the monitoring sites are not at the basin inlet(s) or outlet(s). In fact, estimates for *L*_in_, *L*_out_, *I*_del_, and *I*_acc_ can be obtained for basins within the model area that lack monitoring data altogether. Another advantage is that estimates for both *I*_del_ and *I*_acc_ can be obtained with the SPARROW model and Equation (1), whereas use of stream-load monitoring data alone allows only for calculation of a net difference between *I*_del_ and *I*_acc_.

## Application of the SPARROW Model to Simulate Dissolved-Solids Transport

The SPARROW model was designed to predict long-term average values of constituent loads that are delivered to downstream receiving waters. The model is statistically based and explains constituent loads in relation to upstream sources and watershed properties, such as soil characteristics, climate conditions, and land cover, which influence the transport of constituents to streams and their delivery to receiving water bodies. SPARROW models are typically used to predict constituent loads in streams, from both small and large watersheds, and to identify the primary sources of the constituents, especially in unmonitored watersheds. The SPARROW model relates the dependent variable, defined as the annual dissolved-solids load transported out of a given stream reach of the network, to several explanatory variables that reflect upstream environmental conditions: source variables, land-to-water delivery variables, and reach-loss variables. Source terms reflect the annual mass of dissolved solids released from point and nonpoint sources. These sources are either attenuated or amplified by land-to-water delivery processes, which reflect environmental conditions of the land surface that affect release of dissolved solids from sources and delivery to streams. The product of the source and land-to-water delivery terms reflects the annual dissolved-solids mass that is released from these sources and delivered to the streams. Reach-loss terms are applied to the annual dissolved solids delivered to the streams from the reach catchment and from upstream reaches, and as applied in this study, they reflect environmental conditions that reduce the instream load of dissolved solids. Digital stream networks, such as the enhanced river-reach file 2.0 (ERF1_2) (Nolan *et al.*, 2002) that was used in this study, consist of digital representations of stream and reservoir reaches linked together in downstream order and provide the spatial framework used by the SPARROW model for tracking downstream transport of dissolved-solids loads from stream headwaters to stream mouths. The average catchment size for the 5,214 reaches in the Southwest from the ERF1_2 network is 250 km^2^. The theoretical and statistical details of the SPARROW model are discussed in further detail by Smith *et al.* (1997) and Schwarz *et al.* (2006).

The SPARROW model was calibrated to median annual dissolved-solids loads for the period 1974-2003 at 315 stream-water-quality monitoring sites. The spatial distribution of the sites (Figure 1) is uneven, generally due to a monitoring bias that favors data collection from streams that serve as reliable water supplies. For example, there are more sites in areas with wet climates and many perennial streams, such as the Upper Colorado River drainage, and fewer sites in areas with dry climates and few perennial streams, such as eastern Nevada and western Utah. Site selection criteria required that the site be on the ERF1_2 network and not redundant to another downstream site on the same reach, have at least 40 water-chemistry samples collected for a period of at least 10 years between 1974 and 2003 at approximately quarterly or more frequent intervals, and have daily discharge data coincident with the period of water-quality record. The second condition allows for adequate representation of variations in dissolved-solids load due to seasonal and climate variations. The water-chemistry (major ion, residue on evaporation, or specific conductance) data and daily discharge data came from the USGS National Water Information System database and were used in a site-specific multiple regression equation that related dissolved-solids concentration to discharge, season, and year. Daily dissolved-solids loads were predicted for the period of water-quality record on the basis of the regression equations and daily discharges, and these were aggregated to annual loads, of which the median was used to estimate the SPARROW model. Additional detail on load determination is discussed in Anning *et al.* (2007).

With a disparity in the record length and period represented, median annual loads for each site were selected over mean annual loads for SPARROW model estimation because they provide a more robust measure of typical conditions for a stream. With different periods of record among stations, mean values may be particularly unrepresentative of typical conditions if the period of record for the site is short and contained one or more wet years with exceptionally high runoff or one or more dry years of exceptionally low runoff. Dampening the variation between adjacent sites on the stream network due to different periods of record is important so that the SPARROW model accounts for changes in load between sites due to the intervening catchment conditions rather than different climate conditions for the sites. An *ex post facto* comparison shows good agreement between the median annual loads used in this study and long-term mean loads estimated for another study using methods described in Schwarz *et al.* (2006) and indicated that the median annual loads were representative of long-term average conditions as needed for input to the SPARROW model (see Supporting Information section for discussion). For model estimation, the median annual loads were weighted inverse proportionally to the percent error of the annual load estimates.

Dissolved-solids source variables tested during model development for statistical significance and inclusion in the final model included the areas of outcrop of the various geologic units, the area of agricultural lands, human populations, and dissolved-solids loads in water imported from outside the basin of interest or concern. A digital map showing bedrock geology of the conterminous U.S. (King and Beikman, 1974; Schruben *et al.*, 1997) and reach-catchment boundaries from the ERF1_2 were used to determine outcrop areas for geologic units in each reach. Due to the regional scale of this data source, each unit represents dissolved solids delivered from subsurface weathering processes occurring in the bedrock and surficial processes occurring in the bedrock outcrops, soils, and streambed sediments that may occur on top of the unit. Altogether, 70 different geologic units were mapped in the Southwest. Conceptually, the area of each geologic unit could be considered as an individual source in the model, each having different values for the source coefficients and delivering different amounts of dissolved solids. Groups of geologic units, however, were aggregated to simplify the model. Geologic units were first split into groups on the basis of general lithology and age: crystalline (plutonic and metamorphic) rocks, felsic volcanic rocks, mafic volcanic rocks, eugeosynclinal rocks, Quaternary basin fill (generally unconsolidated deposits), Tertiary sedimentary rocks, Mesozoic sedimentary rocks, and Paleozoic and Precambrian sedimentary rocks. Tertiary, Mesozoic, and Paleozoic and Precambrian rocks were further divided into low-yield, medium-yield, or high-yield groups of geologic units. This subsequent division of geologic units was accomplished by transferring individual geologic unit from the low-yield group to the high-yield group, one at a time, and then rerunning the model and comparing model diagnostics. Geologic units were reassigned to the high-yield group if as a result of the transfer (1) the source coefficient, *b*, for the low-yield group decreased and the source coefficient for the high-yield group increased; (2) the probability value of the source coefficients remained about the same or decreased; and (3) the *R*^2^ of the model remained about the same or increased as a result of moving a unit from the low-yield group to the high-yield group. If these three conditions were not met, then the geologic unit was kept in the low-yield group. The Supporting Information section of this paper provides a table that summarizes formal geologic formations contained in each group of geologic units used in this study. All of the Southwest's area was classified into one of these 12 groups, therefore covering all geologic sources of dissolved solids. An exception, areas of Quaternary basin fill are considered in a reach-loss variable discussed later in this section.

Areas of cultivated land and pasture land were determined from the National Land Cover Dataset (Vogelmann *et al.*, 2001) and tested for statistical significance as sources of dissolved solids. Cultivated land includes land used to grow row crops, such as corn, soybeans, vegetables, and cotton; small grains, such as wheat, barley, oats, and rice; and fallow areas. By contrast, pasture includes fields with grasses and (or) legumes planted for livestock grazing or for production of seed or hay crops. Cultivated and pasture lands are largely irrigated in the Southwest due to its dry climate. Cultivated land and pasture land source variables largely represent the release of native solutes from the soils as a result of irrigation, and to a lesser extent from soil additives such as fertilizers and soil amendments such as gypsum. Human populations, based on 1990 census data (GeoLytics, Inc., 2001) and urban land (Vogelmann *et al.*, 2001) were also tested as sources of dissolved solids.

Surface-water diversions in the Southwest inherently carry dissolved solids and can result in removal of dissolved solids from streams in one reach catchment, and deliveries to either the same reach catchment, the next downstream reach catchment, or a reach catchment in another accounting unit (a transbasin diversion). The large number of surface-water diversions in the Southwest and the lack of conveyance structures in the topology of the ERF1_2 network required innovative treatment for this mode of transport. Removal of dissolved solids from stream reaches due to diversions was accounted for using the change in reach discharge and percent basin-fill reach-loss variables, which are discussed below. For the first two cases of diversions listed above, the deliveries were not specially treated by the model, which effectively neglects the difference in conveyance location. This introduces uncertainty in the reach location where the diverted dissolved solids may be retained as a result of irrigation and subsequent infiltration to groundwater, and it is, in part, for this reason that the mass-balance results are computed for accounting units rather than for each reach catchment. For the third case, delivery was accounted for by using estimates for the delivery load in the source variable “imported dissolved solids” which were estimated as part of this study and further described in Anning *et al.* (2007).

Land-to-water delivery variables tested during model development for statistical significance and inclusion in the final model included runoff depth, precipitation depth, air temperature, drainage density, soil permeability, and percentage of selected land covers, including forest, shrubland, grassland, barren, transitional, urban, cultivated, and pasture. Data sources for these variables are described in Anning *et al.* (2007). Land-to-water delivery terms were applied to all geologic unit sources to amplify or attenuate the dissolved-solids deliveries from weathered rocks to the streams as affected by processes specific to each land-to-water delivery variable. Their affect on delivery processes associated with the remaining source variables is likely very different from their effect on geologic sources, or even nonexistent as in the case of imported water. Consequently the value of the land-to-water delivery term was set to equal 1 for the nongeologic source terms. Reach-loss variables tested during model development for statistical significance and inclusion in the final model included change in reach discharge, percent Quaternary basin fill, reservoir presence, and reservoir area. The first two variables were applied to all reaches, whereas the last two were applied only to those reaches containing reservoirs. The reach-loss terms were mathematically constructed to result in a number between 0 and 1, therefore maintaining or decreasing the stream load but not increasing it. For change in reach discharge, the reach-loss term was applied to nonreservoir reaches and took the mathematical form of (1 − δ*T*_*i*_), where δ and *Τ* are the vectors of reach-loss coefficients and variables, respectively, associated with losses of dissolved solids in reach *i*. Change in reach discharge, Δ*Q* (unitless), accounts for loss of dissolved solids due to stream diversions or streamflow infiltration and was determined on the basis of reach-discharge data that are included in the ERF1_2 file, and was calculated as follows: 
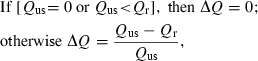
(3)where *Q*_us_ is the sum of stream discharge entering the reach of interest from all adjacent upstream reaches, and *Q*_r_ is stream discharge in the reach of interest (both terms have units of cubic meters per second). For gaining reaches, Δ*Q* is assigned a value of 0, and for losing reaches Equation (2) results in a value for Δ*Q* between 0 and 1. For reaches with no outflow, *Q*_r_ equals 0 and Δ*Q* equals 1. A loss in stream discharge in a reach can occur due to streamflow diversions or due to streamflow infiltration. An implied assumption of Δ*Q* is that changes in dissolved-solids loads across the reach are correlated to changes in streamflow volume across the reach. The reach-loss coefficient for Δ*Q* is expected to be near 1; however, coefficient values larger or smaller than 1 can result because the coefficient does not account for change in dissolved-solids concentrations across the reach due to discharge loss by evaporation or discharge gain by stream inflow. The percent of Quaternary basin fill also accounts for loss of dissolved solids due to stream diversions or streamflow infiltration and reflects additional loss processes not captured by the change in reach-discharge term. These processes probably occur in areas such as pediments or alluvial fans that are not on the reach itself but are in areas of Quaternary basin fill within the reach catchment.

The reservoir presence and reservoir area variables account for retention processes of dissolved solids in reservoirs. Reservoir presence is binary and because dissolved solids generally are considered to behave conservatively in surface-water bodies, the reach-loss coefficient is expected to be small. For reservoir area, the reach-loss term took the mathematical form of 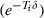 rather than (1 − δ*T*_*i*_) described above. *T*_*i*_ was the surface area of the reservoir in reach *i* (from Nolan *et al.*, 2002), and the reach-loss variable coefficient (δ) was restricted to be >0 to ensure that the term (−δ*T*_*i*_) would be negative and thus represent losses of dissolved solids within reservoirs.

Parametric values for the model coefficients were estimated by using nonlinear least squares (Schwarz *et al.*, 2006). Explanatory variables were “mean-adjusted” whereby the mean value for the explanatory variable was subtracted from each individual observation's value. This adjustment allows for the interpretation of the source coefficients as the mean yield of dissolved solids delivered to streams (Schwarz *et al.*, 2006). Model diagnostics allowed for selection of source terms, land-to-water delivery terms, and reach-loss terms based on optimal accounting for the spatial variation in measured stream dissolved-solids loads. In particular, *t-*test statistics for model coefficients were used to determine the probability of the coefficient being different than 0; those with less than a 0.10 probability level were retained in the model, whereas those with probability levels >0.10 generally were not included. A bootstrap analysis was conducted to assess errors associated with model predictions and to confirm parametric estimation results. The analysis consisted of model reestimation for each of 200 bootstrap iterations, from which population the mean coefficient and upper and lower 90% confidence intervals were determined. Parametric values for the model coefficients were verified by comparing them to the mean value of the coefficients from 200 bootstrap iterations.

In the model application, the coefficients from the parametric estimation were used to predict catchment source loads, reach stream loads, and catchment losses of dissolved solids. Predicted values for these variables are computed in downstream order as the stream load for a given reach is determined as the sum of the stream load generated internally from catchment sources plus the stream-load entering from upstream reaches, minus any reach losses. Where monitored load data were available near the boundary, the model residual for the monitored load was added to the predicted load at the boundary. This adjustment made the mass-balance inflow and outflow estimates reflect monitored loads more closely.

## Model Description

The SPARROW model of dissolved-solids transport for the Southwest contains 15 source variables, 3 land-to-water delivery variables, and 2 reach-loss variables (Table 1). Catchment sources of dissolved solids include 12 geologic units, cultivated land, pasture land, and dissolved solids in imported water. While the source coefficients for eugeosynclinal rocks and low-yield Paleozoic and Precambrian sedimentary rocks were not significant at probabilities less than the 0.10 level (Table 1), they were retained so that the effect of geology for all areas of the Southwest was represented by the model. Significant factors affecting land-to-water delivery include runoff depth, drainage density, and percent barren land. Significant factors related to reach losses included change in reach discharge and percent Quaternary basin fill. The variables included and their functional form in the model are well justified with respect to physical processes affecting transport, and are discussed after a review of the model diagnostics.

**TABLE 1:** Results of Nonlinear Least Squares Estimation and Bootstrap Analysis for the SPARROW Model of Dissolved-Solids Transport in the Southwestern United States.*

		Nonlinear Least Squares Calibration			Bootstrap Analysis			
			
Model Parameters	Coefficient Units†	Coefficient	Standard Error	*p*-Value	Lower 90% Confidence Interval	Mean Coefficient‡	Upper 90% Confidence Interval	*p-*Value
Source variables								
Crystalline rocks	(kg/year)/km^2^	2,284	661	0.001	236	2,195	3,368	0.035
Mafic volcanic rocks	(kg/year)/km^2^	3,733	1,066	0.001	387	3,276	5,216	0.020
Felsic volcanic rocks	(kg/year)/km^2^	5,673	2,473	0.022	-2,167	5,117	8,873	0.060
Eugeosynclinal rocks	(kg/year)/km^2^	21,570	15,800	0.173	-27,860	17,440	45,430	0.165
Sedimentary rocks (kg/year)/km^2^								
High-yield Tertiary	(kg/year)/km^2^	17,400	4,336	<0.001	10,110	17,070	22,520	<0.005
Low-yield Tertiary	(kg/year)/km^2^	10,260	2,163	<0.001	5,083	10,350	13,990	<0.005
High-yield Mesozoic	(kg/year)/km^2^	16,120	5,188	0.002	-5,634	14,560	25,520	0.070
Medium-yield Mesozoic	(kg/year)/km^2^	10,910	5,376	0.043	-2,476	9,768	16,940	0.065
Low-yield Mesozoic	(kg/year)/km^2^	3,025	1,571	0.055	-6,076	1,956	5,304	0.130
High-yield Paleozoic and Precambrian	(kg/year)/km^2^	46,090	19,140	0.017	9,374	43,910	70,170	0.025
Medium-yield Paleozoic and Precambrian	(kg/year)/km^2^	16,480	4,436	<0.001	1,843	16,080	26,590	0.030
Low-yield Paleozoic and Precambrian	(kg/year)/km^2^	1,187	957	0.216	-2,156	697	2,067	0.130
Cultivated land	(kg/year)/km^2^	569,200	151,500	<0.001	222,700	592,500	904,900	0.010
Pasture land	(kg/year)/km^2^	108,700	34,130	0.002	10,380	109,400	188,500	0.025
Imported water	Dimensionless	0.58	0.28	0.043	0.26	0.55	0.80	<0.005
Land-to-water delivery variables								
Runoff depth	(mm/yr)^−1^	14.40	1.85	<0.001	9.78	14.49	20.02	<0.005
Drainage density	(km)^−1^	0.5729	0.1651	<0.001	0.1611	0.5169	0.8666	<0.005
Percent barren land	Dimensionless	0.1106	0.0351	0.002	0.0257	0.1127	0.1912	0.015
Reach-loss variables								
Change in reach discharge	Dimensionless	0.3183	0.1601	0.048	0.0738	0.3250	0.6247	0.025
Percent Quaternary basin fill	Dimensionless	0.0900	0.0488	0.066	0.0112	0.0824	0.1386	0.035
*R*^2^	0.89							
Yield *R*^2^§	0.63							
Mean square error	0.50							
Root mean square error	0.71							
Number of observations	315							

* From Anning *et al.* (2007); bootstrap analysis consisted of 200 calibration iterations.

† Dependent variable in tons per year.

‡ Also called the bootstrap estimate.

§ Meausures reduction in variation of contaminant yield rather than variation in contaminant load (Schwarz *et al.*, 2006).

**FIGURE 2:**
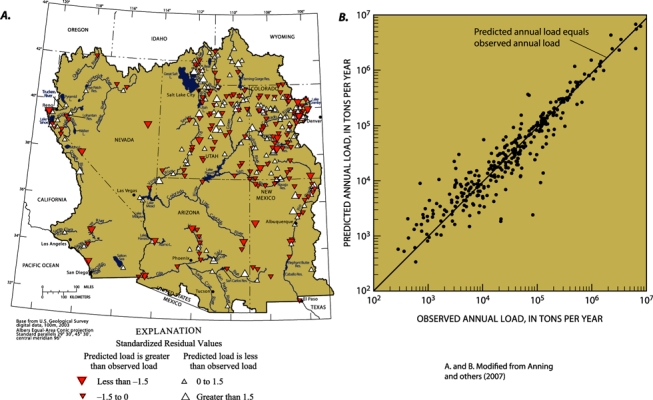
Diagnostic Plots for the SPARROW Model of Dissolved-Solids Transport in the Southwestern United States. (A) Map of standardized residuals. (B) Predicted *vs*. observed annual dissolved-solids loads.

Standard diagnostics indicate that the model is generally unbiased and captures most of the processes affecting dissolved-solids delivery, transport, and loss from rivers in the Southwest. The *R*^2^ value indicates that the model accounts for about 89% of the variability observed in the annual stream-load data (Table 1). The *R*^2^ values tend to be large for SPARROW models partly because much of the variation in the dependent variable is associated with the size of the drainage area upstream from the monitoring sites. The yield *R*^2^ value is 0.63, and reflects the percent variability accounted for in the observed stream loads by the SPARROW model after the variability in the observed data resulting from drainage area is removed (Schwarz *et al.*, 2006). Standard errors of prediction were determined from the 200 estimation iterations of the bootstrap analysis; the average standard error for the 5,214 reaches was 59% of the predicted load. The Shapiro-Wilks test (Shapiro *et al.*, 1968) indicated that the model residuals were normally distributed. The predicted against observed plot shows that residuals are evenly distributed with respect to observed values – residuals are not biased for certain ranges of observed values, nor does the variance of residuals change across the range of observed values (Figure 2). On a broad scale, the residuals generally lack spatial patterns and indicate that the model is spatially unbiased (Figure 2). One feature obvious in Figures 1 and 2, however, is the lack of data in certain basins, including the Little Colorado River Basin in northeastern Arizona, the western part of the Great Salt Lake and Sevier River Basins in western Utah, and most of the central Nevada and eastern California desert basins. It is uncertain whether load predictions in these areas are biased due to lack of representation by monitoring site data for model estimation. Serial correlation of residuals along streams is mitigated because of the way the model estimation treats nested basins and avoids the cascading of errors down a river basin (Smith *et al.*, 1997).

## Interpretations of Model Coefficients

Interpretations of the SPARROW model coefficients (Table 1) provide insight to how specific environmental conditions affect the delivery, transport, and losses of dissolved solids in streams of the Southwest. The coefficients for each geologic source indicate the average annual load of dissolved solids delivered to streams for a given area of that unit under average conditions for the land-to-water delivery variables. Many of the source-coefficient bootstrap confidence intervals of geologic units do not overlap each other, which indicates that the delivery rates of dissolved solids to reaches vary significantly for those rock types, given that all other conditions are equal (Table 1). Negative values of source coefficients for the lower bootstrap confidence intervals are likely a result of some model estimations having one or more source coefficients that are overestimated and the source with a negative coefficient is effectively compensating for that overestimate by reducing the catchment delivery. Source coefficients for geologic units account for both surface processes that deliver dissolved solids through precipitation runoff, and subsurface processes that ultimately deliver dissolved solids to the streambed through groundwater discharge.

Crystalline rocks, which are primarily granitic or metamorphic rocks, deliver dissolved solids to the streams within their catchments at a rate of 2,284 (kg/year)/km^2^ (Table 1). With the exception of low-yield Paleozoic and Precambrian sedimentary rocks, this is the lowest rate among the rock types and reflects the resistance of these rocks to weathering processes. The source coefficient for mafic volcanic rocks is 3,733 (kg/year)/km^2^, which is less than that for felsic volcanic rocks, 5,673 (kg/year)/km^2^. Sedimentary rock types generally are more susceptible to weathering and deliver more dissolved solids than crystalline and volcanic rocks; their source coefficients range from 1,187 (kg/year)/km^2^ for low-yield Paleozoic and Precambrian sedimentary rocks to 46,090 (kg/year)/km^2^ for high-yield Paleozoic and Precambrian sedimentary rocks (Table 1). Sedimentary rocks deposited in a given geologic era have varying source coefficients for dissolved solids. For example, geologic units grouped in the “high-yield Tertiary sedimentary rocks” deliver about 70% more than “low-yield Tertiary sedimentary rocks.”

Qualitatively, the coefficients for geologic sources from this study agree reasonably well to weathering rates determined by other studies. A robust quantitative comparison of the source coefficients to published weathering rates for different rock types generally is precluded by significant differences between the studies. These include differences in the aggregation of rock types and (or) geologic formations reported on by the study, the ions used to assess weathering, climate conditions represented in the study area, and general study approaches. A qualitative comparison, however, can be made by normalizing dissolved-solids yields determined by each stream-chemistry study to the yields for crystalline rocks (Meybeck, 1987).

Relative yields for this study are most comparable to Kenney *et al.* (2009), who used similar geologic units in their SPARROW model for the Upper Colorado River. Differences occur between the two sets of relative yields because this study encompasses a larger study area, the two SPARROW models have somewhat different sets of explanatory variables, and for Kenney *et al.* (2009) the relative yield was normalized to both crystalline and volcanic rocks rather than just crystalline rocks, which effectively lowers the relative yields for other geologic units for their study.

For this and other stream-chemistry studies, the general order of increasing relative yields is crystalline (plutonic and metamorphic) rocks, volcanic rocks, and sedimentary rocks (Table 2). Of the sedimentary rocks, sands, sandstones, and quartzite are generally the least susceptible to weathering, and in cases have smaller relative yields than crystalline rocks (Amiotte Suchet *et al.*, 2003, for example, in Table 2). In this study, low-yield Paleozoic and Precambrian sedimentary rocks yield about half the dissolved solids as do crystalline rocks (Table 2), and while most sedimentary rock types are represented in this group, many formations consist of quartzite. Sandstones, such as those in the Dakota Sandstone, Morrison Formation, and Glen Canyon Group are contained within the low-yield Mesozoic sedimentary rocks, which have a source coefficient about 1.3 times that of crystalline rocks (see Supporting Information section for list of selected formations in each geologic unit in the SPARROW model). Relative yields for shales are generally greater than those of sandstones, but less than carbonate rocks (Table 2). While shales are contained in most of the sedimentary geologic units used in the SPARROW model, some yield much larger amounts of dissolved solids compared to crystalline rocks, such as the Mancos Shale that is contained in the high-yield Mesozoic sedimentary rocks (relative yield of 7.1 in Table 2). The Mancos Shale, along with associated soils derived from it and used for cultivation of irrigated crops and pasture, is a significant source of dissolved-solids in the Upper Colorado River Basin (Iorns *et al.*, 1965; Tuttle and Grauch, 2009). Relative yields for carbonate rocks are larger than sandstones and shales, and are about 12-35 times that of crystalline rocks for other studies (Table 2). In the Southwest, much of the carbonate rock is contained in the medium-yield and in the high-yield Paleozoic and Precambrian sedimentary rocks, which have relative yields of 7.2 and 20.2, respectively. Four of the largest point sources of dissolved solids to the Colorado River (U.S. Department of the Interior, 2005) are springs associated with medium-yield Paleozoic sedimentary rocks in the SPARROW model. These include Glenwood Springs and Dotsero Springs, which discharge solutes from the Eagle Valley Evaporite to the Colorado River (Iorns *et al.*, 1965; Chafin and Butler, 2002); Paradox Springs, which discharge solutes from the Hermosa Formation to the Dolores River (Iorns *et al.*, 1965; Chafin, 2002); and Blue Springs, which discharges solutes from the Redwall Limestone to the Little Colorado River (Metzger, 1961; Bills *et al.*, 2007). Gypsum and halite beds in the Southwest generally occur in geologic formations within the medium-yield and high-yield sedimentary rocks, and in other studies that focus on those specifically, they have the highest relative yields (40 and 80 in Table 2, respectively, from Meybeck, 1987).

**TABLE 2:** Comparison of Relative Yields of Dissolved Solids Due to Chemical Weathering and Erosion for Different Lithologies as Determined From Selected Studies of Stream Chemistry.*

This study, Anning *et al.* (2007)		Kenney *et al.* (2009)		Meybeck (1987)		Amiotte Suchet *et al.* (2003)	
			
Rock Type	Relative Yield	Rock Type	Relative Yield	Rock Type	Relative Yield	Rock Type	Relative Yield
Crystalline rocks	1.0	Crystalline and volcanic rocks	1.0	Granite, gneiss, and mica schists	1	Crystalline (shield) rocks	1.0
Mafic volcanic rocks	1.6			Miscellaneous metamorphic	5	Basalts	5.3
Felsic volcanic rocks	2.5			Gabro	1.3	Acid volcanic rocks	1.7
Eugeosynclinal rocks	9.4			Volcanic rocks	1.5		
*Sedimentary rocks*		*Sedimentary rocks*		*Sedimentary rocks*		*Sedimentary rocks*	
High-yield Tertiary	7.6	High-yield Tertiary	8.1	Sandstones	1.3	Sands and sandstones	0.8
Low-yield Tertiary	4.5	Low-yield Tertiary	3.8	Shales	2.5	Shales	6.2
High-yield Mesozoic	7.1	High-yield Mesozoic	9.4	Carbonate rocks	12	Carbonate rocks	11.8
Medium-yield Mesozoic	4.8			Gypsum	40		
Low-yield Mesozoic	1.3	Low-yield Mesozoic	0.6	Rock salt	80		
High-yield Paleozoic and Precambrian	20.2	High-yield Paleozoic and Precambrian	5.7				
Medium-yield Paleozoic and Precambrian	7.2						
Low-yield Paleozoic and Precambrian	0.5	Low-yield Paleozoic and Precambrian	0.3				
Notes: Used SPARROW model calibrated to stream loads for basins of mixed drainage areas in the Southwestern United States with mixed geology. Analysis based on cations and anoins.		Notes: Used SPARROW model calibrated to stream loads for basins of mixed drainage areas in the Upper Colorado River Basin with mixed geology. Analysis based on cations and anoins. Rock units are similar but not identical to Anning *et al.* (2007).		Notes: Compared stream loads from small river basins in France with uniform lithology. Analysis based on cations and anions, except atmospherically derived CO_2_.		Notes: Assimilated weathering rates based on authors’ previous work and that of Meybeck (1987), and applied to global distribution of outcrops for relative importance of lithologies.	

Gaillardet *et al.* (2003)		Horton *et al.* (1999)		Bluth and Kump (1994)	Stallard and Edmond (1983)
			
Rock Type	Relative Yield	Rock Type	Relative Yield	Order of Increasing Relative Yield	Order of Increasing Relative Yield
Crystalline rock	1	Granitic gneisses	1	1. Sandstone	1. Highly intensely weathered sediments
Volcanics and volcaniclastics	4.1	Andesite	4.9	2. Granite	2. Siliceous terraines
				3. Basalt4. Shale5. Carbonate	3. Marine sediments, including those with carbonates, minor evaporites, and reduced shales
					4. Massive evaporites
*Sedimentary rocks*		*Sedimentary rocks*			
Shales and blackshales	2.3	Carbonates	35.0		
Carbonates	24				
Notes: Comparison of stream loads from river basins within Canada; incorporates those investigated by Millot *et al.* (2002, 2003). Analysis based on cations only. Reported above are rock type averages for several streams reported by Gaillardet *et al.* (2003).		Notes: Comparison of stream loads from the Yellowstone River Basin. Analysis based on cations only.		Notes: Comparison of stream loads in several small river basins in the United States, Puerto Rico, and Iceland. Analysis based on bicarbonate and silica. Data not available for reporting relative yields.	Notes: Comparison of stream chemistry in several small river basins within the Amazon Basin. Analysis based on cations and anions. Data not available for reporting relative yields.

* Relative yield rates are normalized by dividing rate reported for each rock type by the rate for crystalline or related rocks to facilitate comparison.

The source coefficients indicate that the yield of dissolved solids from cultivated land, 569,200 (kg/year)/km^2^, is more than five times greater than that for pasture land, 108,700 (kg/year)/km^2^. The difference in the two source coefficients implies that differences in the type and intensity of farming activities (tillage, crop selection and rotation, and irrigation practices, etc.) and (or) the availability of solutes in the soils associated the two types of agricultural lands yields different dissolved-solids deliveries. Note that these coefficients reflect average conditions for the Southwest, and there is likely local variation due to different irrigation, tillage, and crop rotation practices, as well as differences in the salt content of the soils and parent geologic materials they overlay. Direct comparison of these coefficients cannot be made to those for the geologic units because land-to-water coefficients were not applied to them.

The source coefficient for imported dissolved solids of 0.58 indicates that about 58% of the annual dissolved-solids mass imported to a reach catchment are delivered to the stream and about 42% remains in the catchment, most likely as a result of water uses that do not return the used water to the stream. Imported water carrying the dissolved solids is used, in part, for municipal purposes in several of the major population centers, such as the Los Angeles, Las Vegas, Phoenix, and Tucson metropolitan areas. The loads of dissolved solids contributed by municipal water use are, therefore, partially accounted for by the dissolved solids in the imported water. In areas where imported water is not used, dissolved-solids contributions from municipal water use are not accounted for by the model. While population and urban land were tried as source variables to represent municipal water use, their coefficients were found insignificant, and therefore, not included in the model.

Source loadings from geologic sources were adjusted by three land-to-water delivery variables – runoff depth, drainage density, and percent barren land (Table 1). The positive sign for the runoff-depth coefficient indicates that source loads of dissolved solids from a given geologic unit increase with an increase in runoff depth. This makes physical sense because where runoff depth is greater, there is more precipitation to chemically weather and transport geologic materials to streams. The high level of significance for the coefficient originates from the fact that the SPARROW model predicts load, a product of streamflow and concentration. The spatial variation of runoff depth is correlated to that for precipitation, which is shown in Figure S2. Precipitation, like depth of runoff, was also highly significant in exploratory models. For the final model, however, runoff depth was chosen over precipitation because multicolinearity between variables was smaller using runoff depth. The positive sign for the drainage-density coefficient (Table 1) indicates that source loads of dissolved solids from a given geologic unit increase with an increase in drainage density. This makes physical sense because a denser stream-drainage network would expedite delivery of water and dissolved solids to the streams. The percentage of barren land reflects vegetation density and soil exposure, and conceptually, a decrease in vegetation density and an increase in soil exposure would expedite dissolution of salts from geologic materials. Model results support this interpretation, as a larger percentage of barren lands in a catchment results in a higher delivery of dissolved solids from geologic sources. The percentage of barren land within a catchment also may serve to provide fine adjustments to the runoff depth because more vegetation would impede runoff, and less vegetation would expedite runoff.

Instream transport of dissolved solids through each reach was reduced by two reach-loss variables – the change in reach discharge and percent Quaternary basin fill. Neither of the reservoir retention variables was found significant in developing the SPARROW model and, therefore, they are not included in the final model (Table 1). The lack of significance of these variables confirms the conservative behavior of dissolved solids in reservoirs. The two reach-loss variables reflect processes in which dissolved-solids loads in streams are attenuated because water is removed from the stream; this result is a considerable contrast to reach-loss variables in nutrient SPARROW models, where constituent mass is lost due to biological activity or chemical transformation.

**FIGURE 3:**
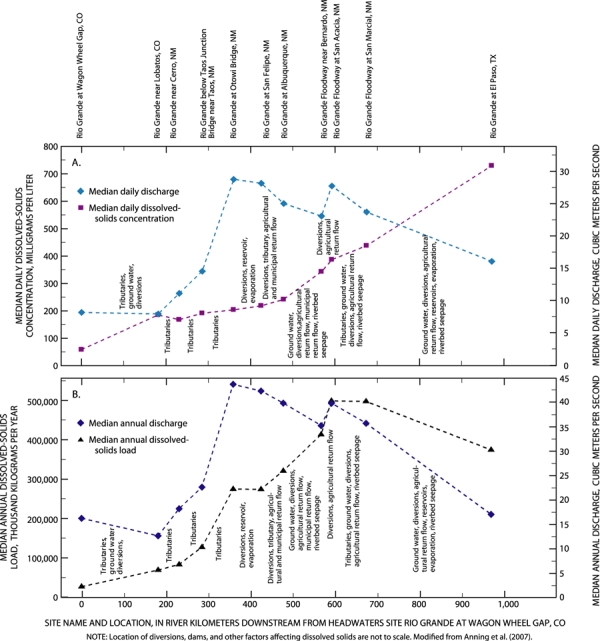
(A) Median Daily Dissolved-Solids Concentrations and Discharge; (B) Median Annual Dissolved-Solids Loads and Discharge, and Factors That Can Affect Concentrations of Dissolved Solids and Loads, for Surface-Water-Quality-Monitoring Sites in the Main Stem of the Rio Grande.

The change in reach-discharge variable was constructed such that instream loads are reduced only when a streamflow loss across the reach occurs. The physical interpretation for the change in reach-discharge coefficient of 0.3183 is that reductions in stream loads are only about one-third the reductions in stream discharge. The smaller reduction in stream load likely results from increases in dissolved-solids concentrations across the reach, which is common for most streams in the Southwest (Figure 3) (figures 17-25 in Anning *et al.*, 2007). Such increases in concentrations can result from evapotranspiration of water by irrigated crops or by riparian vegetation, reservoir evaporation, or from seepage of high concentration groundwater to streams, as shown for the Rio Grande (Figure 3).

For early exploratory models estimated during the model development, the area of Quaternary basin fill was considered as a source variable; however, source coefficients were negative, indicating that the effect of this geologic unit is opposite to that of the other units and reflects a reach loss rather than a source. For the final model, the area of Quaternary basin fill was used as a reach-loss variable, but expressed as a percentage of the total catchment area. Several (99) of the catchments are underlain entirely by Quaternary basin fill, and overall, that unit makes up 22% of the land surface in the Southwest (Figure 1). The coefficient of 0.0900 (Table 1) indicates that for a catchment underlain entirely by Quaternary basin fill, the stream load would be reduced by 9%. The stream-load reduction for catchments with Quaternary basin fill is likely a result of the infiltration of flow in the reach delineated in the ERF1_2 network and all the tributaries to the reach within the reach catchment. Given the latter of the two reasons for the reduction, the percentage of Quaternary basin-fill variable also performs the function of a land-to-water delivery variable.

## Predicted Catchment Delivery Rates

**FIGURE 4:**
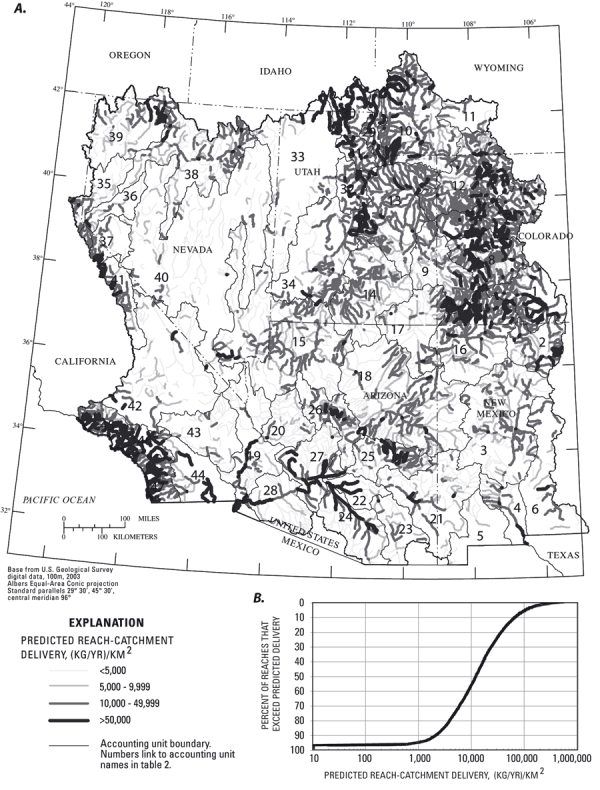
Area-Normalized Predicted Reach-Catchment Delivery Rates of Dissolved Solids to Streams in the Southwestern United States. (A) Spatial distribution; (B) statistical distribution.

The SPARROW model of dissolved-solids transport was applied to 5,214 stream reaches in the Southwest (Figure 4A). Area-normalized reach-catchment delivery rates of dissolved solids to streams ranged from <10 (kg/year)/km^2^ for catchments with dry climates, low-yield geologic units, and no cultivated or pasture lands, to 563,000 (kg/year)/km^2^ for catchments that were almost entirely cultivated land where water from irrigation accelerates the weathering processes (Figures 1 and 4B). The middle 90% of the rates, however, occur in a much narrower range between 1,000 and 107,000 (kg/year)/km^2^ (Figure 4B).

Many of the catchments with delivery rates >50,000 (kg/year)/km^2^ are in the Upper Colorado River Basin or the Upper Rio Grande Basin within Colorado (Figure 4A). In general, the delivery rates in those catchments are high due to the presence of sedimentary rocks, cultivated land, and pasture land in that area (Figure 1; Table 1), as well as high precipitation runoff rates (Gebert *et al.*, 1987). These geologic, land-use, and climatic conditions occur as well in several catchments in the Upper Bear, Lower Bear, Ventura-San Gabriel Coast, Santa Ana, and Laguna-San Diego Coastal accounting units where catchment delivery rates are also high (Figure 4A; Table 3).

**TABLE 3:** Summary of SPARROW Model Predictions Aggregated to Hydrologic Accounting Units of the Southwestern United States.*

						Percentage of Total Internal Deliveries			
						
Hydrologic Accounting Unit	Map #†	Delivery Rate, (kg/year)/km^2^ × 1,000	Accumulation Rate, (kg/year)/km^2^ × 1,000	Ratio of Imports to Internal Deliveries	Ratio of Delivery to Accumulation Rate	Crystalline and Volcanic Rocks	Sedimentary Rocks	Cultivated Lands	Pasture Lands
Upper Rio Grande Basin									
Rio Grande headwaters	1	39	35	0.0	1.1	23	16	42	19
Upper Rio Grande	2	17	7	0.1	2.3	13	62	18	8
Rio Grande - Elephant Butte	3	8	3	0.0	2.6	8	65	22	5
Rio Grande - Caballo	4	19	29	0.0	0.7	3	13	80	4
Mimbres‡	5	4	4	0.0	1.0	21	44	26	9
Rio Grande closed basins‡	6	18	18	0.0	1.0	1	55	39	4
Colorado River Basin									
Colorado headwaters	7	72	<1	0.0	>206	7	69	14	11
Gunnison	8	51	<1	0.0	>146	14	41	31	14
Upper Colorado -Dolores	9	13	2	0.0	7.6	2	75	15	9
Upper Green	10	22	3	0.0	7.9	2	77	7	14
Great Divide closed basin‡	11	5	5	0.0	1.0	0	96	4	0
White-Yampa	12	26	8	0.0	3.3	4	57	30	9
Lower Green	13	22	<1	0.0	>62	0	78	10	13
Upper Colorado - Dirty Devil	14	9	15	0.0	0.6	3	90	3	4
Lower Colorado - Lake Mead	15	13	2	0.3	7.2	8	82	3	7
Upper San Juan	16	23	9	0.0	2.6	8	58	19	14
Lower San Juan	17	14	9	0.1	1.5	0	36	56	8
Little Colorado	18	8	1	0.0	7.3	5	89	4	3
Lower Colorado	19	20	17	0.0	1.2	5	5	70	19
Bill Williams	20	4	4	0.0	1.1	51	25	20	5
Upper Gila	21	10	4	0.0	2.5	13	16	68	3
Middle Gila	22	102	69	0.2	1.5	1	1	96	2
San Pedro-Willcox	23	7	2	0.0	2.7	7	38	43	11
Santa Cruz	24	35	21	0.2	1.7	1	3	93	3
Salt	25	24	9	0.4	2.7	10	61	28	1
Verde	26	11	6	0.0	2.0	19	70	9	3
Lower Gila-Agua Fria	27	49	107	0.2	0.5	2	1	94	3
Lower Gila	28	17	50	1.3	0.3	7	4	77	12
Great Basin and Mojave Desert									
Upper Bear	29	39	15	0.0	2.6	0	54	26	20
Lower Bear	30	151	86	0.0	1.8	0	7	81	12
Weber	31	47	4	0.0	12.3	1	51	35	12
Jordan	32	9	6	0.1	1.4	2	42	20	36
Great Salt Lake‡	33	19	57	0.0	0.3	1	16	72	10
Escalante Desert-Sevier Lake‡	34	13	13	0.0	1.0	11	46	7	36
Truckee‡	35	8	5	0.0	1.8	62	24	0	13
Carson‡	36	9	13	0.4	0.7	27	26	0	46
Walker‡	37	12	12	0.0	1.0	21	45	0	34
Humboldt‡	38	9	9	0.0	1.0	10	40	10	40
Black Rock Desert‡	39	12	12	0.0	1.0	17	16	26	42
Central Nevada Desert Basins‡	40	3	3	0.0	1.0	28	55	1	16
Mono - Owens Lakes‡	41	21	12	0.0	1.8	29	62	0	9
Northern Mojave‡	42	8	9	0.1	0.9	13	24	42	21
Southern Mojave‡	43	4	4	0.0	0.9	52	25	18	5
Salton Sea‡	44	52	247	3.8	0.2	1	5	73	21
Southern California Coastal Basins									
Ventura-San Gabriel Coast	45	41	–§	1.7	–§	5	29	59	7
Santa Ana	46	69	–§	0.5	–§	4	9	83	4
Laguna-San Diego Coastal	47	43	–§	0.3	–§	4	15	75	6
All accounting units						7	37	44	12

* Data compiled from tables 19 and 20 in Anning *et al.* (2007).

† Map # links accounting unit name to number shown on map in Figure 4a.

‡ Closed basin with no natural outflow.

§ Accumulation rate and ratio of deliveries to accumulation were not computed because of unquantified loads carried in treated municipal wastewater that is released to the ocean.

Many of the catchments with lower delivery rates, <5,000 (kg/year)/km^2^, are in the Basin and Range physiographic province (Figure S1) (Fenneman and Johnson, 1946) in Nevada, western Utah, southeastern California, southern and western Arizona, and southwestern New Mexico (Figure 4A). The physiography of this province is largely controlled by normal faulting and contains many uplifted mountain ranges that are separated by long, linear, down-dropped valleys. In general, the low delivery rates in these catchments are a result of the mountains consisting largely of crystalline and volcanic rocks, which have low dissolved-solids delivery coefficients; the valleys being filled with Quaternary basin deposits, which reduce dissolved-solids loads as a reach-loss variable; the lack of cultivated or pasture lands; and the low precipitation runoff rate for that area (Figure 1) (Gebert *et al.*, 1987).

Predicted loads at accounting unit boundaries provided estimates for *L*_in_ and *L*_out_ in Equation (2), and reach-catchment delivery predictions, aggregated by accounting unit, provided estimates for *I*_del_. These estimates, along with point estimates for *T*_imp_ and *T*_exp_ allowed for computation of *I*_acc_ and facilitate the discussion on important source and accumulation areas, important sources, and transport through major river systems as discussed in following sections.

## Important Source and Accumulation Areas

**FIGURE 5:**
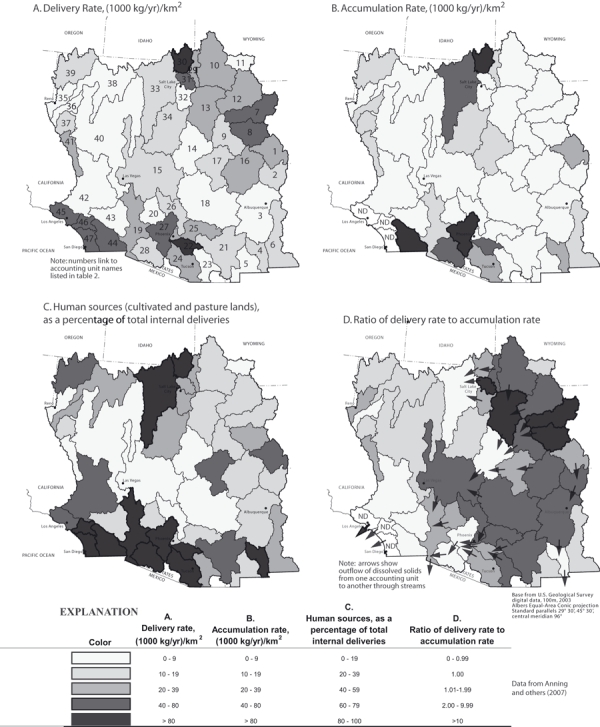
Maps of (A) Delivery Rate, (B) Accumulation Rate, (C) Human Sources, and (D) Ratio of Delivery Rate to Accumulation Rate for Accounting Units in the Southwestern United States.

Delivery rates of dissolved solids from internal sources in the accounting units have considerable variability within the Southwest, ranging from 3,000 (kg/year)/km^2^ for the Central Nevada Desert Basins to 151,000 (kg/year)/km^2^ for the Lower Bear (Table 3; Figure 5A). The median delivery rate was 17,000 (kg/year)/km^2^. Delivery rates were >40,000 (kg/year)/km^2^ in the Colorado headwaters, Gunnison, Middle Gila, Lower Gila-Agua Fria, Lower Bear, Weber, Salton Sea, Ventura-San Gabriel Coast, Santa Ana, and Laguna-San Diego Coastal accounting units. These 10 accounting units, which comprise approximately 11% of the area, are among the most important source areas of dissolved solids in the Southwest. Delivery rates were low – <10,000 (kg/year)/km^2^– in about 30% (14 of the 47) of the accounting units (Table 3; Figure 5A). Delivery rates tend to be greater in accounting units with wetter climates and more runoff (Figure S2), such as the Rio Grande headwaters, Colorado headwaters, and Upper Bear accounting units, and in areas of intensive cultivation of crops and pasture, such as the Salton Sea, Middle Gila, and Lower Bear accounting units. Delivery rates tend to be low in accounting units with drier climates (Figure S2) and where minimal cultivation of crops and pasture occur, such as the Mimbres, Little Colorado, and Central Nevada Desert Basins accounting units.

Accumulation rates of dissolved solids from internal sources in the accounting units also have considerable variability within the Southwest and ranged from <1,000 (kg/year)/km^2^ for the Colorado headwaters, Gunnison, and Lower Green accounting units to 247,000 (kg/year)/km^2^ for the Salton Sea accounting unit (Table 3; Figure 5B). The accumulation rate for the Salton Sea accounting unit was more than twice as large as the second highest rate, 107,000 (kg/year)/km^2^ for the Lower Gila-Agua Fria accounting unit. The median accumulation rate was 9,000 (kg/year)/km^2^. Accumulation rates were >40,000 (kg/year)/km^2^ for the Middle Gila, Lower Gila-Agua Fria, Lower Gila, Lower Bear, Great Salt Lake, and Salton Sea accounting units (Table 3; Figure 5B). These six accounting units are among the most important accumulation areas in the Southwest. Accumulation rates were low – <10,000 (kg/year)/km^2^– in about 55% (26 of the 47) of the accounting units (Table 3; Figure 5B). Accumulation rates tend to be greater in accounting units with closed-surface drainage with substantial inflow such as the Great Salt Lake, significant diversions for irrigation of cultivated and pasture lands such as the Lower Gila-Agua Fria and Lower Bear, or both of the above, as is the case for the Salton Sea. Accumulation rates are smallest in accounting units without substantial inflow or diversions for irrigation of cultivated and pasture lands, such as the Central Nevada Desert Basins. The Ventura-San Gabriel Coast, Santa Ana, and Laguna-San Diego Coastal accounting units are different from the other accounting units in the Southwest in that they each have numerous exports of dissolved solids out of the accounting unit, namely releases of wastewater-treatment plant effluent and reverse-osmosis brine disposal to the Pacific Ocean. These exports are not accounted for within the ERF1_2 stream network and lack of information on the exported loads precluded computation of the accumulation rates for these accounting units.

## Relative Importance of Different Sources

Predictions from the SPARROW model were used to determine the relative importance of the various natural and human-related internal sources of dissolved solids that are delivered to river systems in accounting units of the Southwest. Important internal sources of dissolved solids vary by accounting unit as a result of variation across the Southwest in the area and source coefficients of each geologic unit, cultivated land, and pasture land, which are coarsely shown in Figure 1, and the coefficients and values for each of the land-to-water delivery variables. Geologic units, which represent natural sources of dissolved solids, contribute 44% of the total internal deliveries for all accounting units in the Southwest (Table 3). Dissolved-solids loads associated with cultivated and pasture lands, which comprise about 2.5% of the land area, result from human activities such as irrigation and contribute the remaining 56% of the total internal deliveries for all accounting units in the Southwest.

Crystalline and volcanic rocks contribute 7% of the internal deliveries in the Southwest overall, and contribute over half of the internal deliveries to the Bill Williams, Truckee, and Southern Mojave accounting units (Table 3). Sedimentary rocks are a more important source, contributing 37% of the internal deliveries in the Southwest. In 40 of the 47 accounting units, internal deliveries from sedimentary rocks are greater than those from crystalline and volcanic rocks. Sedimentary rocks contribute over half of the internal deliveries to 19 accounting units, many of which are in the Upper Colorado River Basin (Table 3) and (or) on the Colorado Plateau (see Figure S1 for Physiographic Province location). Natural sources – crystalline, volcanic rocks, and sedimentary rocks – contribute over half of the internal deliveries of dissolved solids in 27 of the 47 accounting units (Table 3).

Through the acceleration of weathering processes by irrigation, cultivated lands contribute 44% of the internal deliveries in the Southwest overall, and contribute over half of the internal deliveries to 14 accounting units (Table 3). While pasture lands contributed less than half of the internal deliveries in all accounting units, this source combined with cultivated lands contributed half or more of the internal deliveries in 20 accounting units (Table 3). The percentage of internal deliveries from cultivated and pasture lands is particularly high in accounting units in the southern parts of California, Arizona, and New Mexico, where precipitation is low and therefore deliveries from natural sources are also low, and where temperatures are warm and conducive to cultivating crops (Figure 4C).

The discussion above has focused on deliveries from sources internal to the accounting units. For some accounting units, deliveries from imported dissolved solids are also important. For the Lower Gila and Ventura-San Gabriel Coast accounting units, dissolved solids contributed from imported sources are greater than contributions from internal sources, and those for the Salton Sea are about 3.8 times as large as dissolved solids delivered from internal sources (Table 3, ratio of imports to internal deliveries). Other accounting units, however, receive a more moderate amount of deliveries from imported dissolved solids and include the Lower Colorado-Lake Mead, Middle Gila, Santa Cruz, Salt, Lower Gila-Agua Fria, Carson, Santa Ana, and Laguna-San Diego Coastal accounting units.

## Regional-Scale Transport

The hydraulic connection of accounting units in the Southwest by river systems and by long artificial conveyance systems allows for transport of dissolved solids over considerable distances from source areas to accumulation areas. In the regional context of dissolved-solids transport in the Southwest, the accounting units generally can be classified as either net sources, net sinks, or isolated basins on the basis of the ratio of the accounting unit delivery rate to the accumulation rate. Many accounting units behave as source-type basins in which more dissolved solids are delivered to surface waters than are accumulated from retained surface waters (*I*_del_ ≫ *I*_acc_) (Table 3; Figure 5D). For these accounting units, excess dissolved solids that were not retained (accumulated) were transported to other accounting units as outflow or exports. Four of these accounting units – the Colorado Headwaters, Gunnison, Lower Green, and Weber – delivered more than 10 times the amount of dissolved solids than accumulated.

By contrast, nine accounting units behaved as net sinks, in that accumulation rates were greater than delivery rates (*I*_acc_ ≫ *I*_del_). These included the Rio Grande-Caballo, Upper Colorado-Dirty Devil, Lower Gila-Agua Fria, Lower Gila, Great Salt Lake, Carson, Northern Mohave, Southern Mohave, and Salton Sea accounting units (Table 3; Figure 5D). These accounting units accumulated the dissolved-solids mass generated internally as well as that imported from other accounting unit streams or through imported dissolved solids. Note that while the last five accounting units listed are topographically closed basins from which natural outflow is not possible, the first four listed are drained by streams; however, because a substantial amount of water is consumed before leaving the accounting unit, the dissolved solids are not flushed through to the next downstream accounting unit. For eight accounting units, delivery and accumulation rates were approximately the same and their ratio was equal to 1.0 because they are closed basins that had neither outflow nor exports (Table 3; Figure 5D).

Areas with high accumulation rates are likely accumulating dissolved solids in the subsurface where flow infiltrates streambeds or where agricultural or urban irrigation water is applied and infiltrates. Accumulation can occur as salts precipitated in the soil or underlying sediments, and (or) dissolved salts in soil-pore or sediment-pore water, or groundwater. This accumulation represents a water-quality concern with respect to dissolved-solids concentration in groundwater in areas where concentrations of dissolved solids in the infiltrating streamflow or irrigation water are higher than the concentration of the receiving groundwater. In areas where concentrations of the infiltrating stream water or irrigation water are lower than the concentration of dissolved solids in the receiving groundwater, the infiltrating water would serve to dilute the receiving groundwater.

## Considerations for Use of Study Results

When using the results from this study, it is important to remember that the purpose of this study was to understand at a regional scale, what the sources, transport, and sinks of dissolved solids are in the southwestern U.S. Results from this study contain considerable uncertainty at the local scale, but do show for the region where the major sources and sinks of dissolved solids are. Smaller, accounting unit scale studies are recommended for refinement of these results, especially where there were particularly high deliveries or accumulation rates of dissolved solids, or where there are few monitoring sites to constrain predictions. Site-scale studies remain important parts of the science that helps understand processes at the local scale. Understanding such processes can help define the importance of regional-scale explanatory input datasets needed for models such as the SPARROW model, as well as help define the model structure from a mathematical perspective.

When using the results from the SPARROW model, it is important to keep in mind that the model was calibrated to conditions across the whole southwestern U.S. Consequently, each model coefficient represents average rates for processes affecting the delivery, transport, and accumulation of dissolved solids across the study area. In reality, these rates vary spatially from catchment to catchment. In addition, the coefficients represent lumped conditions for processes. For example, a given geologic unit in the model represents many different geologic formations. The relative makeup of these geologic formations for the modeled geologic unit will vary from catchment to catchment that contain that geologic unit. Consequently, the delivery of dissolved solids from each geologic formation will vary spatially, but only a single delivery rate is represented by the source coefficient for the geologic unit in the SPARROW model. Similarly, the model applies a single source coefficient for cultivated lands across the whole study area. In reality, dissolved-solids deliveries from cultivated lands are, in part, a function of the irrigation, tillage, and crop rotation practices, as well as the salt content of the soils and geologic formations they overlay, and because these naturally vary, so do deliveries from cultivated lands. Strategically, SPARROW models should be developed with input datasets where the dissolved-solids (or other constituent) response to the input variable is homogenous across the study area; however, such variables are rarely available in practice. Smoothing of predicted SPARROW catchment deliveries so as to align predicted stream loads with those observed at monitoring sites has the effect of accounting for the discussed local reach-to-reach scale variation in response to a given explanatory variable. While the smoothing made for more realistic stream loads and transport of the dissolved solids, the resulting uncertainty of the model predictions was not assessed.

When using results from the mass balance (Equations 1 and 2), it is important to keep in mind that the terms in the balance show changes in dissolved-solids mass within the accounting unit river systems. This is especially relevant for consideration of the total accumulation of dissolved solids in the soils and groundwater. Model results only show the potential for accumulation in the soils and groundwater resulting from surface-water sources of dissolved solids. Accumulation in soils and groundwater from non-present-day surface-water sources of solutes is not considered in this mass balance. For example, application of water (i.e., artificial recharge from irrigation or for aquifer replenishment) to previously undisturbed lands may mobilize salts previously accumulated in soils and the unsaturated zone over the millennia and transport the solutes to the aquifer.

## Geographical Summary and Conclusions for Management Strategies

A SPARROW model of dissolved solids transport in streams of the Southwest was developed by using as input several source variables (type and extent of surficial geologic units, area of agricultural lands, and amount of imported dissolved solids), land-to-water delivery variables (runoff depth, drainage density, and percent barren land), and reach-loss variables (change in reach discharge and percent Quaternary basin fill). Areas with particularly high deliveries of dissolved solids to stream reaches were shown in Figure 4, and represent areas to investigate salinity-control opportunities. Model predictions were used in a mass-balance approach to determine the sources, transport, and accumulation of dissolved solids. Areas with significant accumulation were shown in Figure 5, and represent areas where groundwater quality may be adversely affected by dissolved solids. Geographic differences in the sources, transport rates, and accumulation rates pose different salinity problems and management strategies for different areas.

In the Upper Rio Grande (numbers 1-4 in Figure 5A), dissolved-solids delivery rates in accounting units decrease in the downstream direction until the Rio Grande-Caballo accounting unit is reached, where delivery rates are greater and human sources contribute 84% of the internal deliveries. Accumulation of dissolved solids is high in the Rio Grande headwaters accounting unit (35,000 (kg/year)/km^2^) because of internal losses within the northern part of this accounting unit. Because part of this area is topographically closed, it retains dissolved solids. In addition, substantial diversions for agriculture are made in this unit, which removes water from streams, and only part of the diverted dissolved-solids load returns to the streams in irrigation-return flows. Further downstream, accumulation in the Rio Grande-Caballo accounting unit is also high due to diversions for agriculture. Accumulation in the Mimbres and Rio Grande closed basins (accounting units 5 and 6 in Figure 5A) is not high compared to other accounting units in the Southwest, despite the fact that they are closed to surface drainage; for these accounting units the accumulation rate equals the delivery rate.

Most of the accounting units in the Great Basin and Mojave Desert (numbers 29-44 in Figure 5A), which do not drain to the ocean, have low dissolved-solids delivery rates as a result of the lack of agriculture (Figure 1) and the low precipitation rates for that region. Where agriculture does occur, such as in the Lower Bear, Great Salt Lake, and Salton Sea accounting units, human sources are greater than natural sources. With the exception of the Great Salt Lake and Salton Sea, accumulation rates are equal or nearly equal to delivery rates for these accounting units owing to their closed-basin topography. The Great Salt Lake and Salton Sea accounting units have much greater accumulation rates than delivery rates of dissolved solids as a consequence of receiving substantial inflow of imported dissolved solids from adjacent accounting units. Accumulation in these accounting units may adversely affect groundwater supplies.

Accounting units in the Colorado River Basin above the Upper Colorado-Dirty Devil (numbers 7-13 in Figure 5A) have some of the highest delivery rates for internal deliveries of dissolved solids in the Southwest, and some of the lowest accumulation rates (Table 3). Consequently, the large loads of the dissolved solids delivered to streams in these accounting units flow to downstream accounting units in southern Arizona and California where substantial diversions occur. While the largest source of dissolved solids in these upper basin accounting units is sedimentary rocks, salinity control of both natural and human sources is important because of the large magnitude of deliveries and their transport to downstream accounting units.

Downstream from the Upper Colorado-Dirty Devil accounting unit (numbers 15 and 19 in Figure 5A), most of the Colorado River is diverted and exported to the southern parts of Arizona, California, and Nevada (22, 24-28, 44-47) for agricultural and municipal uses. Consequently, deliveries of dissolved solids from human sources and transbasin deliveries greatly exceed those from natural sources in several accounting units – specifically the Middle Gila, Lower Gila-Agua Fria, Lower Gila, Santa Cruz, Salton Sea, Ventura-San Gabriel Coast, Santa Ana, and Laguna-San Diego accounting units. In addition, consumption of the water carrying the dissolved solids, along with insufficient drainage or flushing, results in substantial accumulation in most of these same accounting units. For these accounting units, salinity control in upstream accounting units helps reduce accumulation rates in them, and strategies that focus on salt accumulation within the soils, unsaturated zone, and shallow groundwater are also important.

## SUPPORTING INFORMATION

**Data S1.** Assessment on the adequacy of the median annual dissolved-solids loads used in this study for representing long-term average conditions, as well as a description of the geologic units used in the model, and physiographic provinces and climate of the Southwest.

Please note: Neither AWRA nor Wiley-Blackwell is responsible for the content or functionality of any supporting materials supplied by the authors. Any queries (other than missing material) should be directed to the corresponding author for the article.
