# Supplementary material for: Modeled Sources, Transport, and Accumulation of Dissolved Solids in Water Resources of the Southwestern United States
Source: J Am Water Resour Assoc. 2011 Oct;47(5):1087–109. doi: 10.1111/j.1752-1688.2011.00579.x (PMC3307630; doi:10.1111/j.1752-1688.2011.00579.x)
Supplement: Supplementary file 1 [file jawr0047-1087-SD1.doc]

# Supplementary Material

This section provides additional information relevant to the main paper, including an assessment on the adequacy of the median annual dissolved-solids loads used in this study for representing long-term average conditions, as well as a description of the physiographic provinces and climate are available as part of the online paper.

## Physiographic Provinces and Climate

Additional information on physiographic provinces and climate are included here to augment environmental conditions not discussed in the main body of the paper. The Southwest contains eight physiographic provinces (fig S1; Fenneman, 1931), that represent a variety of physiographic features that are largely influenced by geology (table S1). These features include mountain ranges, desert valleys, desert plains, alluvial fans, uplands, high plateaus, and deep canyons. Provinces differ from each other in the assemblage of these features found in each province. Certain physical, chemical, and biological processes tend to be associated with these physiographic features, and as a result, the provinces also are different with respect to hydrology and water chemistry. For this reason, some of the results of this study are referenced to physiographic province.

| **Table S1.** Distinctive features of physiographic provinces and sections in the Southwestern United States | | |
| --- | --- | --- |
| [Data from Fenneman and Johnson, 1946] | | |
| **Physiographic province** | **Physiographic section** | **Distinctive features** |
| Basin and Range | Great Basin | Isolated north-south mountain ranges separated by desert valleys |
|  | Sonoran Desert | Widely separated short mountain ranges within desert plains |
|  | Salton Trough | Desert alluvial fans and delta plain; Gulf of California |
|  | Mexican Highland | Isolated ranges separated by aggraded desert valleys |
|  | Sacramento section | Mature block mountains with gently tilted strata; block plateaus |
| Colorado Plateaus | High Plateaus of Utah | High block plateaus that are locally lava-capped or terraced |
|  | Uinta Basin | Dissected high-relief plateau |
|  | Canyon Lands | Young to mature canyon-carved plateaus with high relief |
|  | Navajo section | Young plateaus with moderate relief |
|  | Grand Canyon section | High block plateaus cut by Grand Canyon |
|  | Datil section | Lava flows and volcanic necks |
| Wyoming Basin | (Not divided into sections) | Elevated dissected plains with isolated low mountains |
| Southern Rocky Mountains | (Not divided into sections) | Complex granitic and sedimentary mountains with intermountain basins; Continental Divide |
| Middle Rocky Mountains | (Not divided into sections) | Complex anticlinal mountains with intermontane basins |
| Cascade - Sierra Mountains | Sierra Nevada | Block mountain ranges tilted west with alpine peaks on east flank; granitic and glaciated |
| Pacific Border | Los Angeles Ranges | Narrow ranges and broad fault blocks; alluviated lowlands |
| Lower Californian | (Not divided into sections) | Dissected westward-sloping granitic upland |

The Southwest generally has abundant sunshine, moderate to high wind, low relative humidity, a large range in daily temperature, and a semiarid climate. Winter precipitation is derived from eastward-tracking Pacific storms and generally is greater than summer precipitation, especially in mountainous areas. Winter precipitation generally falls from widespread storms, whereas summer precipitation falls from spatially scattered thunderstorms. Variations in climate across the Southwest (figs. S2 and S3; Daymet, 2006) are largely affected by altitude, proximity to the coast, and physiographic features such as mountains.


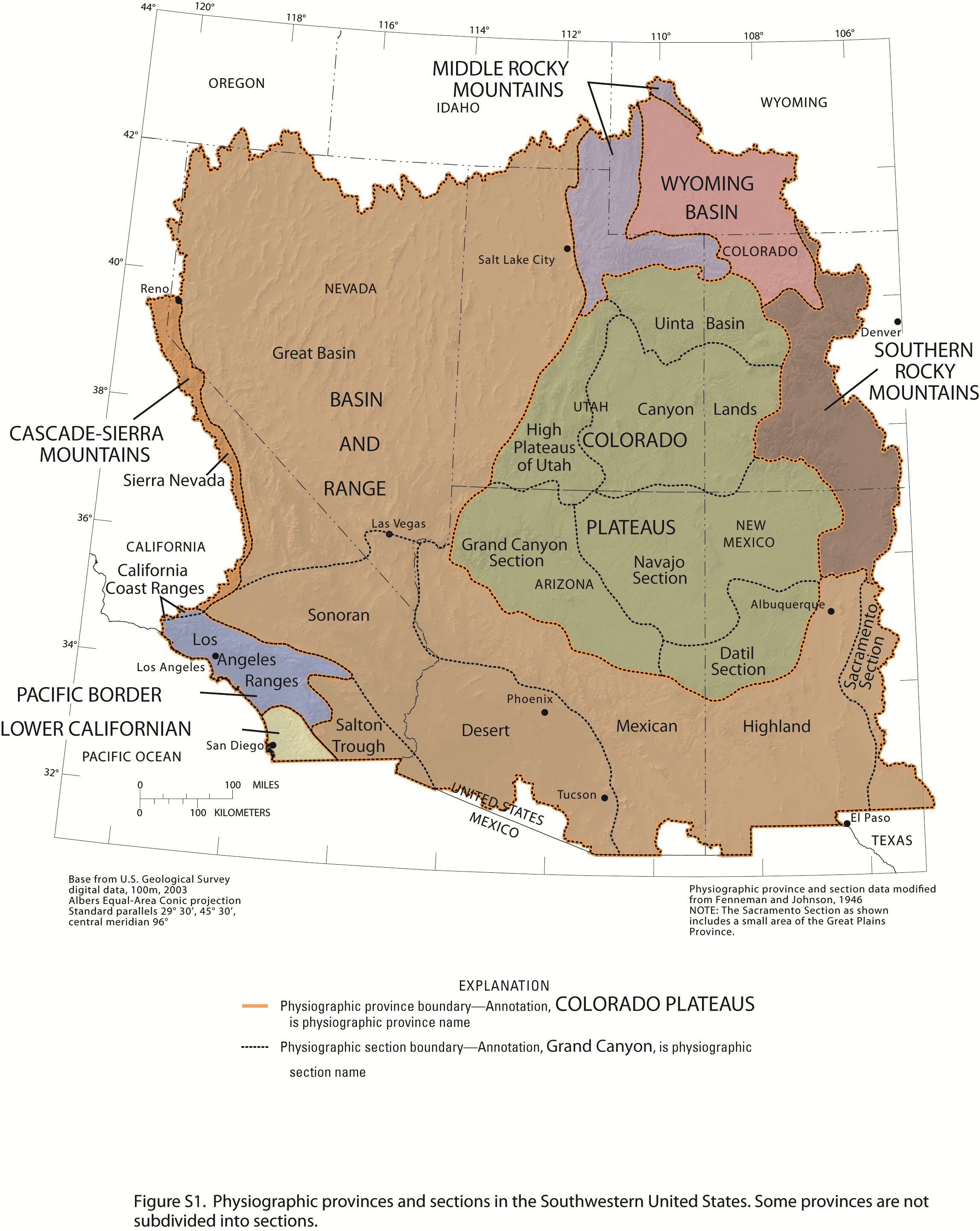


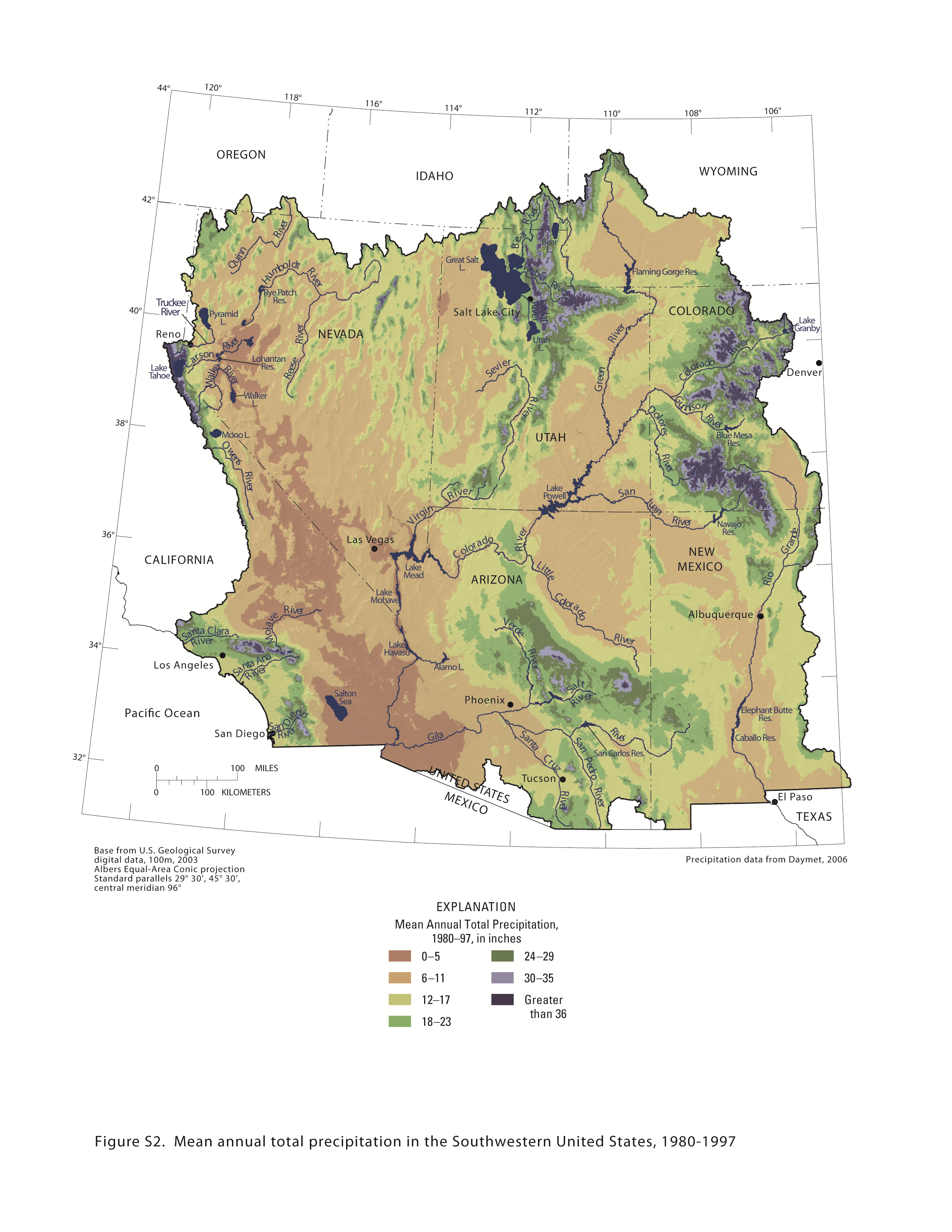


***
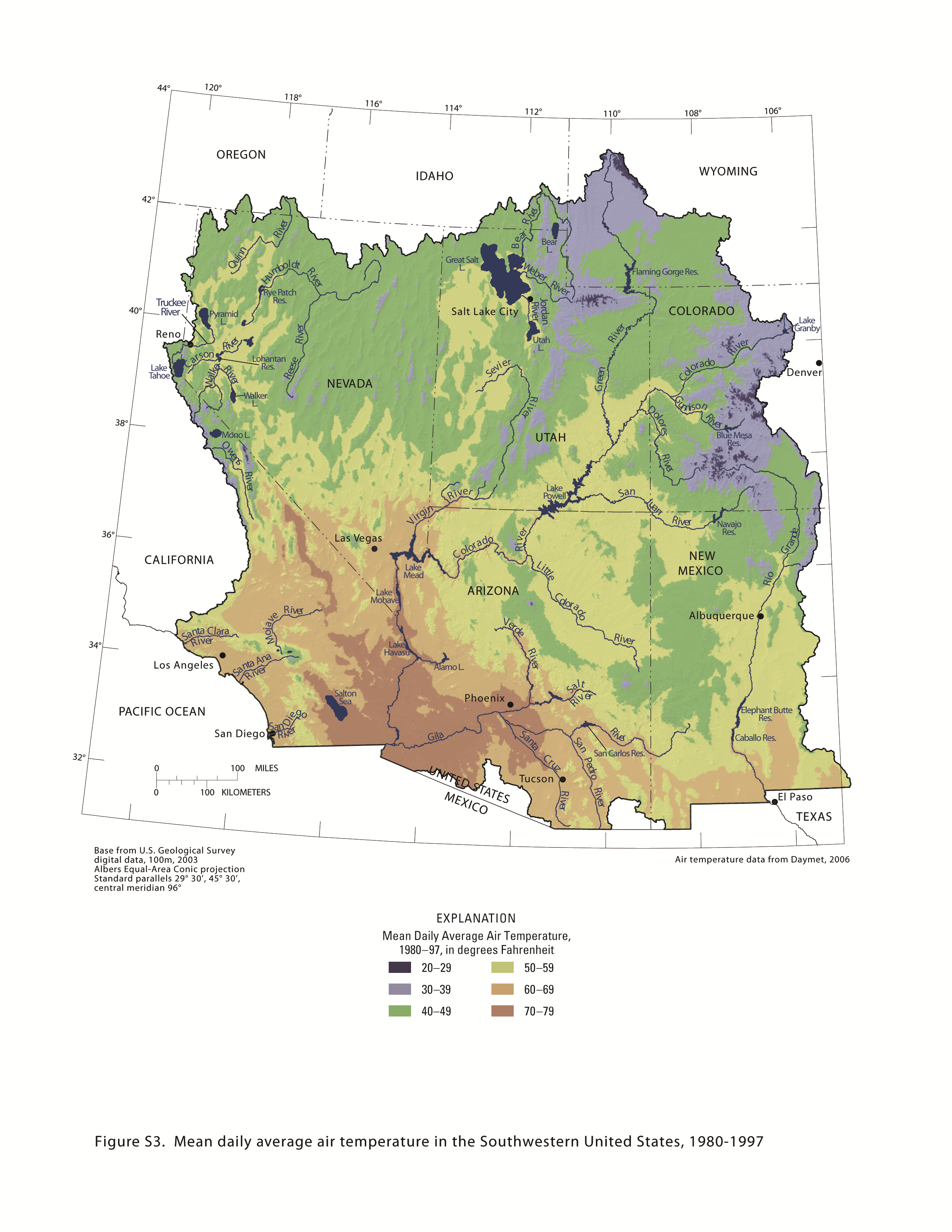
***

## Geologic Units

Geologic units used in the SPARROW model were compiled from King and Beikman (1974), which are often unfamiliar to those investigating basin and site-scale studies. Consequently, a table was made correlating geologic units used in the model to King and Beikman (1974) units and to selected formally defined geologic formations comprising those units (table S2).

| **Table S2.** Groups of geologic units used in the SPARROW model of dissolved-solids transport in the Southwestern United States | | | |
| --- | --- | --- | --- |
| [Modified from Anning et al. (2007). Geologic units are from King and Beikman (1974); AZ, Arizona; CA, California; CO, Colorado; NM, New Mexico; NV, Nevada; UT, Utah; WY, Wyoming; Gp, Group; Fm, Formation; mi, mile] | | | |
| **King and Beikman (1974) geologic units** | | | **Noninclusive summary of rocks and geologic units depicted in state geologic maps that generally correspond to the King and Beikman (1974) Map^1^** |
| **Geologic Unit** | **Area, km^2^** | **Name** |
| **Crystalline rocks - 113,100 km^2^ total area** | | | |
| Ti | 6,346 | Tertiary intrusive rocks | Intrusive rocks ranging in composition from granite to diorite in AZ, CA, CO, NM, NV, and UT |
| Kg | 28,490 | Cretaceous granitic rocks | Mostly granite, quartz monzonite, and granodiorite in AZ, CA, and NV |
| Kg_3_ | 2,564 | Latest Cretaceous granitic | Mostly granite, quartz monzonite, and granodiorite in AZ |
| Kg_2_ | 5,232 | Upper Cretaceous granitic | Granite, quartz monzonite, granodiorite, quartz diorite in CA and NV |
| Kg_1_ | 181 | Lower Cretaceous granitic rocks | Granite, quartz monzonite, granodiorite, quartz diorite in CA |
| T_R_g | 2,202 | Triassic granitic group | Granite, quartz monzonite, granodiorite, quartz diorite in CA and NV |
| Jg | 5,879 | Jurassic granitic rocks | Granite, quartz monzonite, granodiorite, quartz diorite in CA and NV |
| Jmi | 311 | Jurassic mafic intrusives | Intrusive rocks ranging in composition from diorite to gabbro in NV |
| Pzg_2_, Pzg_3_ | 440 | Upper Paleozoic granitic rocks | Granites in CA |
| Cg | 52 | Cambrian granitic rocks | Granites in CO |
| Yg_2_,Yg_3_ | 233 | Younger Y granitic rocks | Granites in AZ and NM |
| Yg_1_ | 8,340 | Older Y granitic rocks | Granites and quartz monzonites in AZ, CO, and NM |
| Ya | 285 | Anorthosite | Anorthositic rocks in CA |
| Xg | 15,488 | X granitic rocks | Granites, quartz monzonite, and granodiorites in AZ, CA, CO, and NM |
| Xm | 27,635 | X orthogneiss and paragneiss | Gniess and schist in AZ, CA, CO, NV, and UT |
| X | 6,268 | X metasedimentary rocks | Metasedimentary rocks including quartzite in AZ and NM |
| Wg | 622 | W granitic rocks | Granite and granodiorite in WY |
| Wgn | 2,512 | W orthogneiss and paragneiss | Schist, gniess, and quartzite in UT and WY |
| W | 26 | W metasedimentary rocks | Schist, metagreywacke, and metaconglomerate in WY |
| **Mafic volcanic rocks - 146,600 km^2^ total area** | | | |
| Qv | 20,824 | Quaternary volcanic rocks | Basaltic to andesitic lava flows and associated volcaniclastic sedimentary rocks in AZ, CA, NM, and UT |
| Tpv | 62,238 | Pliocene volcanic rocks | Basaltic to andesitic lava flows and associated volcaniclastic sedimentary rocks in AZ, CA, CO, NM, NV, and UT |
| Tmv | 26,004 | Miocene volcanic rocks | Basaltic to andesitic flows and associated volcaniclastic sedimentary rocks in AZ, CA, CO, NM, NV, and UT |
| lTv | 36,286 | Lower Tertiary volcanic rocks | Basaltic to andesitic flows and associated volcaniclastic sedimentary rocks in AZ, CA, CO, NM, NV, and UT |
| Kv | 673 | Cretaceous volcanic rocks | Basaltic to andesitic flows and associated volcaniclastic sedimentary rocks in AZ and NM |
| lMzv | 622 | Lower Mesozoic volcanic rocks | Various composition flows and tuffs in NV |
| **Felsic volcanic rocks - 62,000 km^2^ total area** | | | |
| Qf | 3,704 | Quaternary felsic volcanic rocks | Rhyolitic to andesitic flows and tuffs in CA and NM |
| Tpf | 20,720 | Pliocene felsic volcanic rocks | Rhyolitic to andesitic flows and tuffs in AZ, NV, and UT |
| Tmf | 29,863 | Miocene felsic volcanic rocks | Rhyolitic to andesitic flows and tuffs in AZ, NM, and NV |
| lTf | 7,718 | Lower Tertiary felsic volcanic rocks | Rhyolitic to andesitic flows and tuffs in CO and NM |
| **Eugeosynclinal rocks - 26,700 km^2^ total area** | | | |
| uMze | 1,114 | Upper Mesozoic eugeosynclinal | Volcaniclastic sedimentary rocks and metavolcanic rocks in AZ, CA, and NV |
| lMze | 12,173 | Lower Mesozoic eugeosynclinal | Volcaniclastic sedimentary rocks and metavolcanic rocks in AZ, CA, and NV |
| T_R_Pe | 2,435 | Triassic and Permian eugeosynclinal | Volcaniclastic sedimentary rocks and metavolcanic rocks in NV |
| uPze | 3,419 | Upper Paleozoic eugeosynclinal | Volcaniclastic sedimentary rocks and metavolcanic rocks in CA, and NV |
| lPze | 7,589 | Lower Paleozoic eugeosynclinal | Volcaniclastic sedimentary rocks and metavolcanic rocks in CA, and NV |
| **Quaternary basin fill - 313,860 kmi^2^ total area** | | | |
| Q | 313,860 | Quaternary | Alluvium, colluvium, lake, playa, terrrace deposits; in some areas young basalt flows (All states). Mostly unconsolidated or semiconsolidated |
| **Low-yield Tertiary sedimentary rocks - 162,900 km^2^ total area** | | | |
| Tp | 1,528 | Pliocene | Various marine units in CA mostly consisting of moderately consolidated sandstone, siltstone, shale, and conglomerate |
| Tpc | 76,871 | Pliocene continental | Alamosa Fm (CO), Bidahochi Fm (AZ and NM), Chuska Sandstone (NM), Gila Gp (AZ and NM), Las Feveras Fm (NM), Santa Fe Gp (NM), Sevier River Fm (UT), Salt Lake Fm (UT), and various sedimentary rock units in CA and NV |
| Tm | 2,435 | Miocene | Various marine units in CA mostly consisting of moderately consolidated sandstone, shale, siltstone, conglomerate, and breccia |
| Tmc | 12,432 | Miocene continental | Browns Park Fm (CO and UT), Fence Lake Fm (NM), Los Pinos Fm (NM), Quemado Fm (NM), and other continental sedimentary rock units (all States) |
| Toc | 4,662 | Oligocene continental | Bishop Conglomerate and Duchesne River Fm (UT), and various continental units in CA mostly consisting of well consolidated sandstone, shale, and conglomerate |
| Te | 1,891 | Eocene | Various marine units in CA mostly consisting of well consolidated shale, sandstone, conglomerate, and limestone |
| Tec | 62,937 | Eocene continental | Baca Fm (NM), Blanco Basin Fm (NM), Bridger Fm (CO and UT), Cub Mountain Fm (NM), El Rito Fm (NM), Fowkes Fm (UT), Galisteo Fm(NM), Hart Mine Fm (NM), Lobo Fm (NM), Love Ranch Fm (NM), San Jose Fm (CO), Sanders Canyon Fm (NM), Skunk Ranch Fm (NM), Timberlake Fm (NM), San Juan Fm (NM), Uinta Fm (UT), Wasatch Fm (CO, UT and WY), Washakie Fm (WY) |
| Tx | 181 | Paleocene | Various marine units in CA mostly consisting of well consolidated sandstone, shale, and conglomerate |
| **High-yield Tertiary sedimentary rocks - 42,740 km^2^ total area** | | | |
| Tel | 25,615 | Eocene lacustrine | Green River Fm (CO, UT, and WY) |
| Txc | 17,120 | Paleocene continental | Clarion Fm (UT), Flagstaff Limestone (UT), Fort Union Fm (CO and WY), Middle Park Fm (CO), and Nacimiento Fm (CO and NM) |
| **Low-yield Mesozoic sedimentary rocks - 95,360 km^2^ total area** | | | |
| uK | 3,652 | Upper Cretaceous | Beartooth Fm (NM), Iron Springs Fm (UT), Hilliard Shale (WY), Pinkard Fm (AZ), Sarten Fm (NM), and various units in CA mostly consisting of sandstone, shale, and conglomerate |
| uK_4_ | 6,061 | Navarro Group | Fruitland Fm (CO and NM), Lance Formation (CO and WY), Lewis Shale (CO and WY), Kirtland Shale (CO and NM), and Pictured Cliffs Sandstone (CO and NM) |
| uK_1_ | 20,668 | Woodbine and Tuscaloosa groups | Dakota Sandstone (AZ, CO, NM, and UT), Cedar Mountain Fm (UT), and Kelvin Fm (UT) |
| J | 36,390 | Jurassic | Morrison Fm and San Rafael Gp (AZ, CO, NM, UT) |
| JT_R_ | 28,594 | Lower Jurassic and upper Triassic | Glenn Canyon Gp (AZ, CO, NM, UT) |
| **Medium-yield Mesozoic sedimentary rocks - 77,200 km^2^ total area** | | | |
| uK_3_,uK_3_a | 29,422 | Taylor Group | Mesa Verde Gp (CO, NM, UT and WY ), Lewis Shale (NM), and Williams Fork Fm (CO) |
| lK,lK_1_,lK_2_ | 5,543 | Lower Cretaceous | Blind Bull Fm (WY), Frontier Fm (WY), Gannet Gp (WY), Hell-to-finish Fm (NM), Mojado Fm (NM), and select units in AZ |
| T_R_ | 42,243 | Triassic | Chinle Fm (AZ, CO, NM, UT), Moenkopi Fm (AZ, CO, NM, UT) |
| **High-yield Mesozoic sedimentary rocks - 64,280 km^2^ total area** | | | |
| uK_2_ | 48,666 | Austin and Eagle Ford Groups | Baxter Shale (WY), Cravasse Canyon Fm (NM), Mancos Shale (AZ, CO, NM, UT), Point Lookout Sandstone (NM), and Tres Hermonos Fm (NM), Mesa Verde Gp (AZ) |
| Kc | 3,600 | Cretaceous continental | Continental sedimentary rocks mostly in NV and AZ and includes units such as the Baseline Sandstone (NV), King Lear Fm (NV), Newark Canyon Fm (NV), and Willow Tank Fm (NV). |
| lMz | 12,018 | Lower Mesozoic | Several units from Nevada including the Auld Lang Syncline and Star Peak Gps; the Augusta Mountain, Cane Spring, Dixie Valley, Gabbs, Luning, Sunrise, and Tobin Favret Fms; and the Aztec Sandstone. Also includes the Stump Fm and Nugget Sandstone (WY) |
| **Low-yield Paleozoic and Precambrian sedimentary rocks - 114,630 km^2^ total area** | | | |
| P_3_b | 259 | Upper part of Guadalupian Series | Artesia Gp (NM) |
| P_3_ | 259 | Guadalupian Series | Queen and Grayburg Fms (NM) |
| P_2_b | 31,676 | Upper part of Leonardian Series | Coconino Sandstone (AZ and UT), Glorieta Sandstone (AZ and NM), Kaibab Limestone (AZ and UT), San Andreas Fm (AZ and NM), and Toroweap Fm (AZ) |
| P_1_ | 855 | Wolfcampian Series | Hueco Fm (NM) |
| P_1_c | 2,797 | Wolfcampian Series continental | Abo Fm (NM) |
| P | 16,576 | Permian | Arcturus Fm (NV and UT), Coconino Sandstone (NV); Cutler Gp (UT), Diamond Creek Sandstone (UT), Gerster Limestone (UT), Hermit Shale (NV), Kaibab Limestone (NV), Kirkman Limestone (UT), Oquirrh Group (UT), Park City Gp (NV and UT), Pequop Fm (NV), Phosphoria Fm (UT), Plympton Fm (UT), Queantoweap Sandstone (NV), Rib Hill Sandstone (NV), and Toroweap Fm (NV); several units in CA mostly consisting of shale, conglomerate, limestone and dolomite, sandstone, slate, hornfels, quartzite, and minor pyroclastic rocks |
| uPzc | 1,684 | Upper Paleozoic clastic wedge facies | Chainman Shale, Diamond Peak Fm, Eleana Fm, Joana Limstone, Mercury Limestone, Narrow Canyon Limestone, and Pilot shale in NV |
| C | 25,667 | Cambrian | Abrigo Fm (AZ), Bloomington Fm (UT), Blacksmith Fm (UT), Bolsa Quartzite (AZ), Bonanza King Fm (NV), Dunderberg Shale (NV), El Dorado Dolomite (NV), Geddes Limestone (NV), Gold Hill Fm (NV), Geertsen Canyon Quartzite (UT), Hamburg Dolomite (NV), Harmony Fm (NV), Langston Fm (UT), Nopah Fm (NV), Nounan Dolomite (UT), Osgood Mountain quartzite (NV), Pioche Shale (NV), Prospect Mountain Quartzite (NV), Secret Canyon Shale (NV), St. Charles Fm (UT), Stirling Quartzite (NV), Tapeats Sandstone (NV), Tonto Gp (AZ), Ute Fm (UT), Windfall Fm (NV), Wood Canyon Fm (NV), Worm Creek Quartzite (UT), and Zabriskie Quartzite (NV); several units in CA mostly consiting of sandstone, shale, limestone, dolomite, chert, quartzite and phyllite |
| lPz | 23,569 | Lower Paleozoic | Caballero Fm (NM), Contadero Fm (NM), Devils Gate Limestone (NV), Dotsero Fm (CO), Dyer Dolomite (CO), Ely Springs Dolomite (NV and UT), Escabrosa Gp (NM), Eureka Quartzite (NV and UT), Gilman Sandstone (CO), Guilmett Fm (NV and UT), Helms Fm (NM), lone Mountain Dolomite (NV); Las Cruces Fm (NM), Laketown Dolomite (NV and UT), Lake Valley Fm (NM), Leadville Limestone (CO), Madison Limestone (CO), Nevada Fm (NV), Onate Fm (NM), Parting Fm (CO), Peerless Fm (CO), Percha Shale (NM), Pilot Shale (UT), Pogonip Gp (NV and UT), Rancheria Fm (NM), Sawatch Fm (CO), Sevy Dolomite (NV and UT), Simonson Dolomite (NV and UT), Sly Gap Fm (NM), Watson Ranch Quartzite (UT); several units in CA and WY mostly consisting of limestone, dolomite, sandstone, and shale |
| Y | 11,292 | Y sedimentary rocks | Apache Gp (AZ), Grand Canyon Super Gp (AZ), Troy Quartzite (AZ), Uinta Mountain Gp (UT); several units in CA consisting of conglomerate, sandstone, shale, limestone, dolomite, marble, and gniess |
| **Medium-yield Paleozoic and Precambrian sedimentary rocks - 48,460 km^2^ total area** | | | |
| uPz | 48,460 | Upper Paleozoic | Alamitos Fm (NM), Amsden Fm (WY), Bird Spring Fm (NV), Callville Limestone (NV), Chainman Shale (UT), Colina Fm (NM), Concha Fm (NM), Cutler Fm (CO), Deseret Limestone (UT), Earp Fm (NM), Ely Limestone (NV), Epitaph Fm (NM), Escabrosa Limestone (AZ), El Paso Limestone (AZ), Flechado Fm (NM), Great Blue Limestone (UT), Hermosa Fm (CO), Humbug Fm (UT), Joana Limestone (UT), La Pasada Fm (NM), Lodgepole Limestone (UT), Madera Fm (NM), Martin Fm (AZ), Minturn Fm (CO), Morgan Formation (CO), Ochre Mountain Limestone (UT), Percha Shale (AZ), Phosphoria Fm (WY), Redwall Limestone (AZ), Rico Fm (CO), Riepe Spring Limestone (NV), Round Valley Fm (CO), Sandia Fm (NM), Temple Butte Limestone (AZ), Weber Sandstone (CO), Woodman Fm (UT); several units in CA mostly consisting of shale, sandstone, conglomerate, limestone, dolomite, chert, hornfels, marble and quartzite |
| **High-yield Paleozoic and Precambrian sedimentary rocks - 23,280 km^2^ total area** | | | |
| P_2_a | 19,114 | Lower part of Leonardian Series | Callville Limestone (AZ), Cutler Gp (AZ), Hermit Shale (AZ), Naco Gp (AZ), Queantoweap Sandstone (AZ), Supai Gp (AZ), and Yeso Fm (NM) |
| Z | 4,170 | Z sedimentary rocks | Black Canyon Fm (UT), Browns Hole Fm (UT), Caddy Canyon Quartzite (UT), Inkom Fm (UT), Kelley Canyon Fm (UT), Maple Canyon Fm (UT), McCoy Creek Gp (NV), Mineral Fork Fm (UT), Mutual Fm (UT), Papoose Creek Fm (UT), Wyman Fm (NV) |
| ^1^Data were compiled by comparison of outcrop location of King and Beikman (1974) units to outcrop locations shown in State geologic maps compiled by: Hintze (1980), Love and Christiansen (1985), Jennings and others (1977), Richard and others (2000), Stewart and others (1978), Scholle (2003), and Tweto (1979). In some cases only some of the geologic units that comprise a given formation or group actually outcrop in the listed states. Not all units, formations, or groups depicted in the state maps as occuring within the outcrop area of geologic units depicted by King and Beikman (1974) are listed here | | | |

## Assessment of median annual dissolve- solids loads

The SPARROW model was designed to simulate long-term average values of water-quality constituent loads. To assess the representativeness of the median annual loads of dissolved solids used in this study and computed in Anning and et al. (2007), loads from 132 of the stations were compared to long-term mean loads computed using the program “Fluxmaster”, which implements the preferred method for computation of long-term mean loads and is described in Schwarz et al. (2007). Methods of determining median annual loads of dissolved solids were previously summarized in this paper and described in detail in Anning et al. (2007).

Differences in the two methods and input datasets may contribute to differences in the two different sets of load estimates, and are therefore summarized here. Anning et al. (2007) used streamflow and water chemistry monitoring data from 1974-2003, whereas the Fluxmaster estimates were based on data from 1980-2009 because these were intended for use in a different study. Anning et al. (2007) used three measures of dissolved-solids concentrations (sum of the solids, residue on evaporation, and specific conductance) and rectified them to make them equivalent to the sum of the solids measure of dissolved solids. For the Fluxmaster estimates, only the sum of the solids data was used. Both methods use regression techniques and relate concentrations and loads to explanatory variables of streamflow, season, and a long-term trend. Anning et al. (2007) specified a parabolic (squared) term for the long-term trend, whereas only a linear trend was specified in the Fluxmaster estimates. Anning et al. (2007) computed annual loads for each year with available data, and then selected the median of those years to represent long-term conditions. The Fluxmaster estimates represent the detrended mean annual load for base year 2000. This estimate can loosely be interpreted as the load that would have been observed in year 2000 had mean flow conditions occurred that year, after accounting for trends that have occurred over time in concentrations, flow, and the relation between concentration and flow.


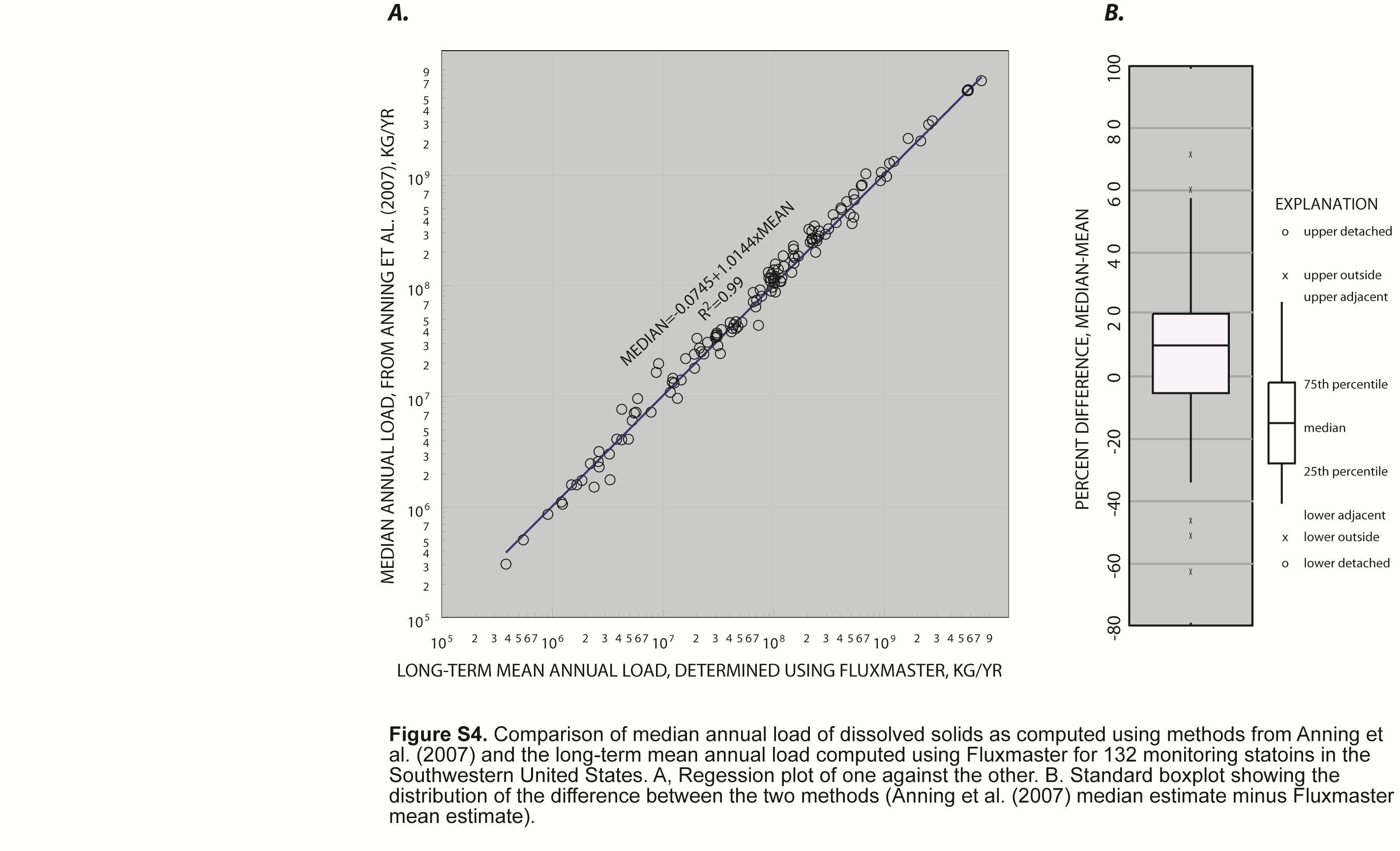


Overall, median annual loads of dissolved solids computed by Anning et al. (2007) compare well to those estimated using Fluxmaster (fig. S4) and therefore are representative of long-term conditions. The slope of a regression between logged (base 10) values for the two datasets is greater than one (1.0144, fig. S4a), indicating that load estimates from Anning et al. (2007) are generally larger than those determined using Fluxmaster, as do calculated percent differences between the two datasets (fig. S4b). The average percent difference is 8.7 %, which indicates a bias in the Anning et al. (2007) estimates to be greater than those estimated using Fluxmaster. Half of the percent differences fall between -5% and 20% (fig. S4b, 25^th^ and 75^th^ percentiles), indicating that for most sites the two estimates are reasonably similar. While some of the bias toward larger load estimates by Anning et al. (2007) is due to the differences in methods and input datasets, a substantial of the bias is likely a result of the Fluxmaster Data set representing a later time frame. Anning et al. (2007) showed that 70.3 percent of the monitoring sites in that study had decreasing trends in flow-adjusted concentrations, and that the mean trend for all sites from 1974-2003 was an 8 percent decrease. Streamflow for most sites has likely been below average conditions as precipitation for the later part of the Fluxmaster data (1999-2009) has generally been less than average in most parts of the study area (National Oceanic and Atmospheric Administration, 2011). Given that the estimates from Fluxmaster were for a later period, estimated loads would be expected to be smaller for the later period due to smaller concentrations and flows. Given that the average percent difference between the two different load estimates is reasonably small, and that the difference is in the direction expected in consideration of observed trends, it is clear that the median annual load estimates from Anning et al. (2007) provide a representative estimate of long-term average conditions and are sufficient for calibration of the SPARROW model.

# Literature Cited

Anning, D.W., 2003. Assessment of selected inorganic constituents in streams in the Central Arizona Basins study area, Arizona and northern Mexico, through 1998. U.S. Geological Survey Water-Resources Investigations Report 03-4063, 116 p. http://pubs.usgs.gov/wri/wri034063/

Daymet, 2006. Daymet daily surface weather and climatalogical summaries: climatalogical summary maps. <http://www.daymet.org/climateSummary.jsp>

Fenneman, N.M., 1931. Physiography of the western United States. New York, McGraw-Hill, 534 p.

National Oceanic and Atmospheric Administration, 2011. National Climatic Data Center, State of the Climate, Annual drought reports for 2003-2009, available at <http://www.ncdc.noaa.gov/sotc/drought/2009/13>.

Schwarz, G.E., A.B. Hoos, R.B. Alexander, and R.A. Smith, 2006. The SPARROW surface water-quality model—Theory, applications, and user documentation. U.S. Geological Survey Techniques and Methods Report, book 6, chap. B3, 248 p. <http://pubs.usgs.gov/tm/2006/tm6b3/contents.htm>.
